# Does working memory training in children need to be adaptive? A randomized controlled trial

**DOI:** 10.1111/cdev.14180

**Published:** 2024-10-23

**Authors:** Regine Cassandra Lau, Peter J. Anderson, Susan Gathercole, Joshua F. Wiley, Megan Spencer‐Smith

**Affiliations:** ^1^ School of Psychological Sciences Monash University Melbourne Victoria Australia; ^2^ Victorian Infant Brain Studies (VIBeS), Clinical Sciences Murdoch Children‘s Research Institute Melbourne Victoria Australia; ^3^ Department of Pediatrics, School of Medicine University of California, Irvine Irvine California USA; ^4^ Center for Newborn Research Children‘s Hospital of Orange County Orange California USA; ^5^ MRC Cognition and Brain Sciences Unit University of Cambridge Cambridge UK; ^6^ Department of Psychiatry University of Cambridge Cambridge UK; ^7^ Psychosocial Oncology Peter MacCallum Cancer Centre Melbourne Victoria Australia

## Abstract

Most cognitive training programs are adaptive, despite limited direct evidence that this maximizes children's outcomes. This randomized controlled trial evaluated working memory training with difficulty of activities presented using adaptive, self‐select, or stepwise compared with an active control. At baseline, immediately, and 6‐months post‐intervention, 201 Australian primary school children (101 males, 7–11 years) completed working memory tests (near and intermediate transfer) and the Raven's Standard Progressive Matrices, and caregivers completed the attention‐deficit/hyperactivity disorder‐Rating Scale‐5 (far transfer). The intervention comprised ten 20‐min sessions delivered in class. For each training condition, compared with the active control, there was no evidence of transfer immediately or 6‐months post‐intervention (negligible to small effects). This trial provides no evidence that adaptive working memory training maximizes children's outcomes.

**Abbreviations**
ADHD‐5‐RS: attention‐deficit/hyperactivity disorder‐Rating Scale‐5 home version
BF: Bayes factor
COMET: COMmon assumptions of cognitivE Training
IMI: Intrinsic Motivation Inventory
IMS: Intrinsic Motivation Scale
SPM: Standard Progressive Matrices

## BACKGROUND

Landmark research suggested it was possible to improve children's working memory performance in the short term using computerized working memory training, resulting in improved fluid reasoning and reduced hyperactive behavior (Klingberg et al., 2002, 2005). Attempts to replicate these findings have shown some evidence for improvements on working memory tasks similar in structure to the training activities (near transfer), but limited evidence for improvements on tasks dissimilar in structure (intermediate transfer) and in other untrained cognitive and behavioral domains (far transfer) (Byrne et al., 2020; Gathercole et al., 2019; Kassai et al., 2019; Sala & Gobet, 2020). Evidence to date suggests that initial training benefits, if any, diminish over time (Melby‐Lervåg et al., 2016; Melby‐Lervåg & Hulme, 2013; Sala & Gobet, 2017).

Computerized cognitive training interventions involve repeated practice on activities targeting one or more cognitive abilities, such as working memory, with the aim of improving the targeted ability (Green et al., 2019). A key design feature of such interventions is an adaptive approach to setting the difficulty of a training activity so that it is constantly adjusted to the trainee's performance. Initially, researchers proposed that the adaptive method worked by challenging the trainee's cognitive limits to induce plasticity (Klingberg, 2010; Lövdén et al., 2010). Consistent with this view, neural connectivity changes in children associated with adaptive working memory training improvements have been observed in select studies (Astle et al., 2015; Jones et al., 2022; Westerberg & Klingberg, 2007), but not all (Kelly et al., 2020, 2021). Furthermore, it has been speculated that by ensuring activities are sufficiently challenging and not too easy or difficult, the adaptive method may be valuable for maintaining trainee motivation (von Bastian & Oberauer, 2014).

Direct evidence that the adaptive method maximizes training outcomes is lacking (see Redick, 2019; von Bastian et al., 2022; von Bastian & Eschen, 2016). Initial support for adaptive cognitive training in children came from studies demonstrating greater training effects and transfer following adaptive training relative to a non‐adaptive control in which training difficulty was fixed at a low level (Dunning et al., 2013; Holmes et al., 2009; Karbach et al., 2015; Klingberg et al., 2002, 2005). Few studies, though, have directly examined the adaptive method alongside other methods for increasing training difficulty compared to a suitable control. To address this gap, von Bastian and Eschen (2016) compared outcomes following working memory training to an active control in adults when training activity difficulty was adjusted using (1) an adaptive approach, (2) a self‐select approach, or (3) a randomized approach where the difficulty of the training activity varied at random and independent of the trainee's performance. Although adults in the adaptive condition outperformed those in the active control condition on the trained activity, this was also the case for the self‐select and randomized conditions. While near transfer effects were not examined, none of the training conditions showed improvements on intermediate or far transfer tasks. Enjoyment, effort, and overall training motivation were similar across the training conditions. The authors concluded that varying the difficulty of a training activity may be sufficient to achieve training effects, regardless of the method used. These findings question the common assumption that an adaptive approach is needed to maximize working memory training outcomes. However, in children, who are the most widely researched population of working memory trainees and whose working memory abilities are actively developing, whether adaptive training is superior or not to non‐adaptive approaches compared to an active control remains to be determined.

Our study aimed to determine if adaptive working memory training in children is superior for a range of outcomes. In a randomized controlled trial of primary school children (7–11 years), we explored adaptive, self‐select, or stepwise approaches to setting the difficulty of training activities, compared with an active control. Near, intermediate, and far transfer outcomes were measured immediately and 6‐months post‐intervention. Although our aim was exploratory, we expected children in the adaptive, self‐select, and stepwise conditions would show improvements on measures of near transfer (but not intermediate or far transfer) immediately post‐intervention but not at 6‐months post‐intervention.

We studied approaches that reflect children's learning experiences in the classroom and may therefore be considered ecological. They share elements identified by theorists as optimal for children's cognitive development and learning, such as providing opportunities to master activities and practice on activities slightly out of their current performance range (see, for example, ‘scaffolding’ and ‘zone of proximal development’; Vygotsky, 1978). The randomized approach used by von Bastian and Eschen (2016) was not explored in our study as it does not clearly reflect a learning experience in the classroom.

## METHODS

This trial is part of the COMmon assumptions of cognitivE Training (COMET) study. Detailed methods are published in the trial protocol (Lau et al., 2023), and key methodologies are summarized below. The trial design and analysis plan were registered retrospectively with the Australian New Zealand Clinical Trials Registry (ACTRN 12621000990820) and approved by Monash University Human Research Ethics Committee (24305) and Melbourne Archdiocese Catholic Schools (1066). Reporting follows CONSORT guidelines (Schulz et al., 2010).

### Participants

Participants were children in Grades 2, 3, 4, or 5 at the participating mainstream primary school in metropolitan Melbourne, Australia, who had caregiver consent and assented to participate. Exclusion criteria were a caregiver‐reported vision impairment that cannot be corrected by glasses, hearing impairment that cannot be corrected by a hearing aid, fine motor impairment, and/or intellectual disability, which would prevent participation in the testing and intervention. Teachers distributed the study invitation packs (a caregiver explanatory statement, consent form, and envelope) to children to take home to their caregivers. Children returned completed consent forms in a sealed envelope to their classroom for the research team to collect. Written child assent was obtained at the start of the baseline child testing session.

**FIGURE 1:**
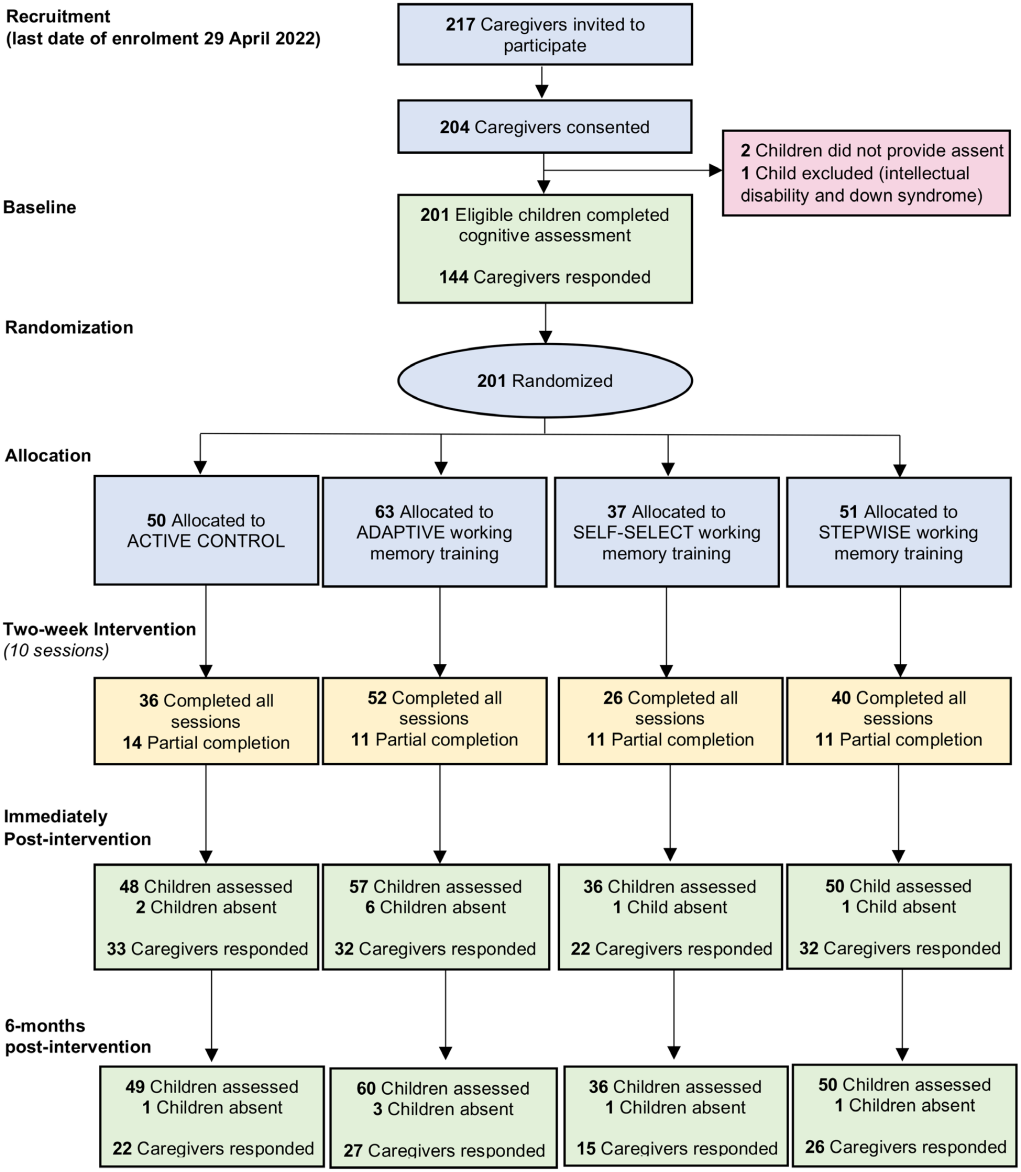
CONSORT diagram of participant flow.

Figure 1 shows the CONSORT diagram. Children were recruited from July 2021 to April 2022. In total, 201 eligible 7‐ to 11‐year‐old children with caregiver consent assented to participate. The consent and assent rate for caregivers and children was 94% and 93%, respectively. Data collection began in February 2022 and ended in December 2022, and was thus achieved within the academic year. Of the 201 children enrolled in the trial, 95% were retained immediately post‐intervention and 97% at 6‐months post‐intervention. Completion rates of all 10 intervention sessions ranged from 70% to 82% across the four conditions.

Power calculations in G*Power (Erdfelder et al., 2009; Faul et al., 2007) indicated 29 children were needed per condition (effect size *f* ^2^ = 0.07; 80% power, *α* = .05) to detect a small to moderate effect of each working memory training condition (adaptive, self‐select, stepwise) compared to the active control on near transfer measures immediately post‐intervention (primary outcome). This study was not powered to detect far‐transfer effects because it was not the primary outcome of interest. A small to moderate effect size was used to calculate power a priori based on reports from meta‐analyses indicating working memory training in children compared with a control produces small to moderate effects on measures classified as near transfer, with moderate effects found on measures using the same paradigm and stimuli as the training activities (Gathercole et al., 2019; Kassai et al., 2019; Melby‐Lervåg et al., 2016; Sala & Gobet, 2020). Thus, our sample size of 36–63 children per condition immediately and 6‐months post‐intervention provided >80% power for each condition.

Table 1 shows the sample characteristics. Demographic characteristics at baseline were similar across conditions, except for age in years, with the self‐select condition being younger, and school level, where there were no Grade 5 children in the self‐select condition. All but one child (in the adaptive condition) spoke predominantly English at home (>80% of the time). The race and/or ethnicity of children and their caregivers were not collected.

**TABLE 1:** Sample characteristics at baseline.

	Active control	Adaptive training	Self‐select training	Stepwise training	Group difference
	(*n* = 50)	(*n* = 63)	(*n* = 37)	(*n* = 51)	*p‐*value
Age in years, mean (SD)	9.15 (1.10)	9.45 (1.22)	8.65 (0.85)	9.11 (1.14)	**.01**
Stratified age, *n* (%)					.24
7 to 8 years	24 (48.0)	23 (36.5)	21 (56.8)	24 (47.1)	
9 to 10 years	24 (48.0)	33 (52.4)	16 (43.2)	24 (47.1)	
11 years	2 (4.0)	7 (11.1)	0 (0.0)	3 (5.9)	
Male sex, *n* (%)	31 (62.0)	34 (54.0)	14 (37.8)	22 (43.1)	.09
School level, *n* (%)					**.02**
Grade 2	14 (28.0)	13 (20.6)	12 (32.4)	14 (27.5)	
Grade 3	13 (26.0)	13 (20.6)	13 (35.1)	13 (25.5)	
Grade 4	13 (26.0)	13 (20.6)	12 (32.4)	12 (23.5)	
Grade 5	10 (20.0)	24 (38.1)	0 (0.0)	12 (23.5)	
Reasoning,^a^ *n* (%)					.35
Below 25th percentile	8 (16.0)	4 (6.3)	3 (8.1)	6 (11.8)	
25th to 75th percentile	30 (60.0)	35 (55.6)	18 (48.6)	24 (47.1)	
Above 75th percentile	12 (24.0)	24 (38.1)	16 (43.2)	21 (41.2)	
Born in Australia,^b^ *n* (%)	32 (86.5)	40 (85.1)	30 (93.8)	34 (89.5)	.67
Living with both parents,^b^ *n* (%)	33 (89.2)	40 (87.0)	31 (96.9)	35 (92.1)	.49
Diagnoses,^b^, ^c^ *n* (%)					.41
ADHD	0 (0.0)	0 (0.0)	0 (0.0)	1 (2.8)	
ASD	1 (2.0)	0 (0.0)	1 (2.7)	0 (0.0)	
Anxiety	0 (0.0)	1 (2.2)	1 (2.7)	0 (0.0)	
Learning difficulty	1 (2.0)	0 (0.0)	0 (0.0)	0 (0.0)	
Elevated hyperactivity and/or inattention,^b^, ^d^ *n* (%)	3 (8.8)	1 (2.3)	4 (12.9)	0 (0.0)	.08
Primary caregiver born in Australia,^b^ *n* (%)	26 (72.2)	31 (67.4)	28 (87.5)	30 (81.1)	.17
Primary caregiver highest level of education,^b^ *n* (%)					.20
Did not complete high school	0 (0.0)	0 (0.0)	0 (0.0)	1 (2.8)	
Completed high school	2 (6.1)	7 (17.1)	2 (6.7)	9 (25.0)	
Completed bachelor degree	22 (66.7)	24 (58.5)	19 (63.3)	14 (38.9)	
Completed post‐graduate degree	9 (27.3)	10 (24.4)	9 (30.0)	12 (33.3)	

*Note*: Significant *p*‐values (<.05) are in bold.

Abbreviations: ADHD, attention‐deficit/hyperactivity disorder; ASD, autism spectrum disorder.

^a^ Raven's Standard Progressive Matrices set A to E; percentiles calculated from normative data from the United States.

^b^ Active control *n* = 37, adaptive training *n* = 46, self‐select training *n* = 32, stepwise training *n* = 38.

^c^ Caregiver reported, and diagnosed by psychologist or general practitioner.

^d^ ADHD‐Rating Scale‐5 total score that has been converted into percentiles.

### Interventions

The Brain Space Program is an experimental intervention designed and developed by our team for the study in Minecraft Education (Mojang Studios and Xbox Game Studios, not an official Minecraft product, not approved by or associated with Mojang). All conditions (adaptive, self‐select, and stepwise working memory training, and active control) were delivered in the same Minecraft environment and had the same motivating features. These included a “space mission” narrative for each training session and the acquisition of experience points that could be used at the end of each session for free play.

The intervention was delivered by teachers in class. Children were to complete 20‐min sessions on each school day for two consecutive weeks (total 10 sessions, total dose 200 min). Children individually performed the intervention on an iPad with headphones to reduce distraction. To account for missed sessions (e.g., absences, public holidays), children could complete up to three training sessions per day during the intervention period. Researchers were on‐site during the intervention period to ensure protocol adherence and compliance and to address any questions teachers or children had about the intervention.

The number of sessions and dose (total training time) varies greatly among child working memory training studies and programs, for example, see meta‐analyses by Melby‐Lervåg et al. (2016) and Sala and Gobet (2020), which report the various training doses of included studies in Supporting Information. Previous small‐sample child working memory training studies with a lower (Loosli et al., 2012: 10 × 6‐min sessions = total dose 60 min) or similar dose (Thorell et al., 2009: 15 × 15‐min sessions = total dose 225 min) to that used in the current study have demonstrated improved outcomes following training.

#### Working memory training conditions

**FIGURE 2:**
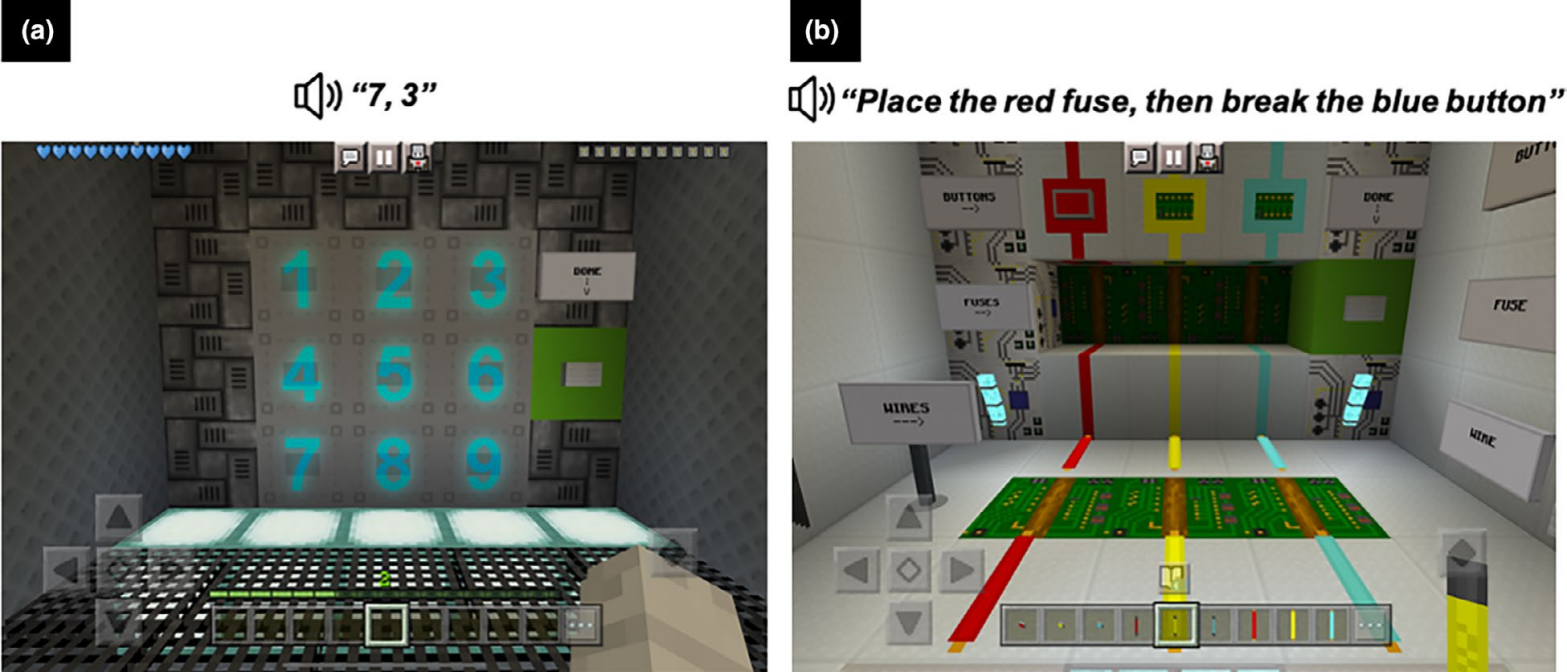
The working memory training activities: (a) backward span and (b) following instructions activities at a span level of 2. See Lau et al. (2023) for more details.

The working memory training consisted of two activities that required the child to temporarily store and manipulate verbally presented information: a backward span activity and a following instructions activity (see Figure 2). In the backward span training activity, a string of digits was presented auditorily, and the child had to remember and immediately recall the sequence of items in reverse order by tapping the digits on the iPad screen. The span level (i.e., difficulty) was increased by increasing the number of digits to be recalled. The following instructions training activity is based on a classroom analog test of working memory developed by Gathercole et al. (2008). The child was introduced to three objects that come in three colors and two actions that require different tapping responses on the iPad screen. The child had to remember and immediately implement an action‐color‐item sequence(s) (e.g., break the red wire, then place the blue button, reflecting a span of two) by using the relevant tapping response on the screen of the iPad with the corresponding color object(s). The span level was increased by increasing the number of action‐color‐item sequences the child had to perform. Each activity comprised five blocks of trials per session, and each block had four trials of a span level (per session: total 20 backward span trials, then 20 following instructions trials).

For the *adaptive* condition, the difficulty of the training activities adapted to the child's performance within each training session on a block‐by‐block basis for the first four blocks (adaptive between block 1 to 2, block 2 to 3, block 3 to 4, and block 5 had the same span as block 4) for each activity. The span increased by one digit/sequence on the next block if the child got three or more trials correct in the block of four trials (≥75% accuracy). The span decreased by one on the next block if the child got two consecutive trials incorrect in the block. Otherwise, the span remained the same. From session two, each training activity started at the span level reached at the end of the previous training session minus one.

For the *self‐select* condition, the child decided at the end of each session if they wanted a training activity to be easier (span level reduced by one digit/sequence), the same or more difficult (span level increased by one digit/sequence) in the next session.

For the *stepwise* condition, the difficulty level increased incrementally across sessions, and this progression differed by age based on current understanding of the development of children's working memory: 7, 8–9, and 10–11 years (Gathercole et al., 2004). The span level increased twice over the training period for all age groups, with the older children progressing faster. In the tenth (final) session, all age groups were training at a backward span level of 4, and a following instructions span level of 3.

#### Active control condition

The active control consisted of new creative building and discovery activities each session. The child created a new room or structure by placing blocks in a grid‐like matrix (analogous to LEGO building in the real world) and explored the 3D virtual space environment. The creative activities were selected based on a review of current activities in Minecraft that primary school teachers use in the classroom that do not target working memory. Importantly, the activities were embedded in the same Minecraft environment as the working memory training activities, and therefore the condition may be considered a placebo (Simons et al., 2016).

### Measures

Table 2 summarizes the measures administered at each time point and identifies the primary endpoint and primary outcome measures.

**TABLE 2:** Trial measures.

Domain/measure	Respondent	Baseline	Immediately post‐intervention	6‐Month post‐intervention
Near transfer				
Backward span digits	Child	•	•^a^	•
Following instructions objects	Child	•	•^a^	•
Backward span letters	Child	•	•	•
Following instructions letters	Child	•	•	•
Intermediate transfer				
2‐back objects	Child	•	•	•
Far transfer				
Raven's SPM sets A to E	Child	•		
Raven's SPM sets A and B	Child		•	•
ADHD‐5‐RS	Caregiver	•	•	•
Child characteristics				
Participant information questionnaire	Caregiver	•	^b^	^b^
Child motivation				
Intrinsic Motivation Scale	Child	•		
Intrinsic Motivation Inventory	Child		•	

*Note*: “•” indicates the measure was administered at this time point.

Abbreviations: ADHD‐5‐RS, attention‐deficit/hyperactivity disorder‐Rating Scale‐5 home version; SPM, Standard Progressive Matrices.

^a^ Primary outcome.

^b^ Caregivers who did not complete the participant information questionnaire at baseline were asked to complete it at later time points.

#### Near transfer

**FIGURE 3:**
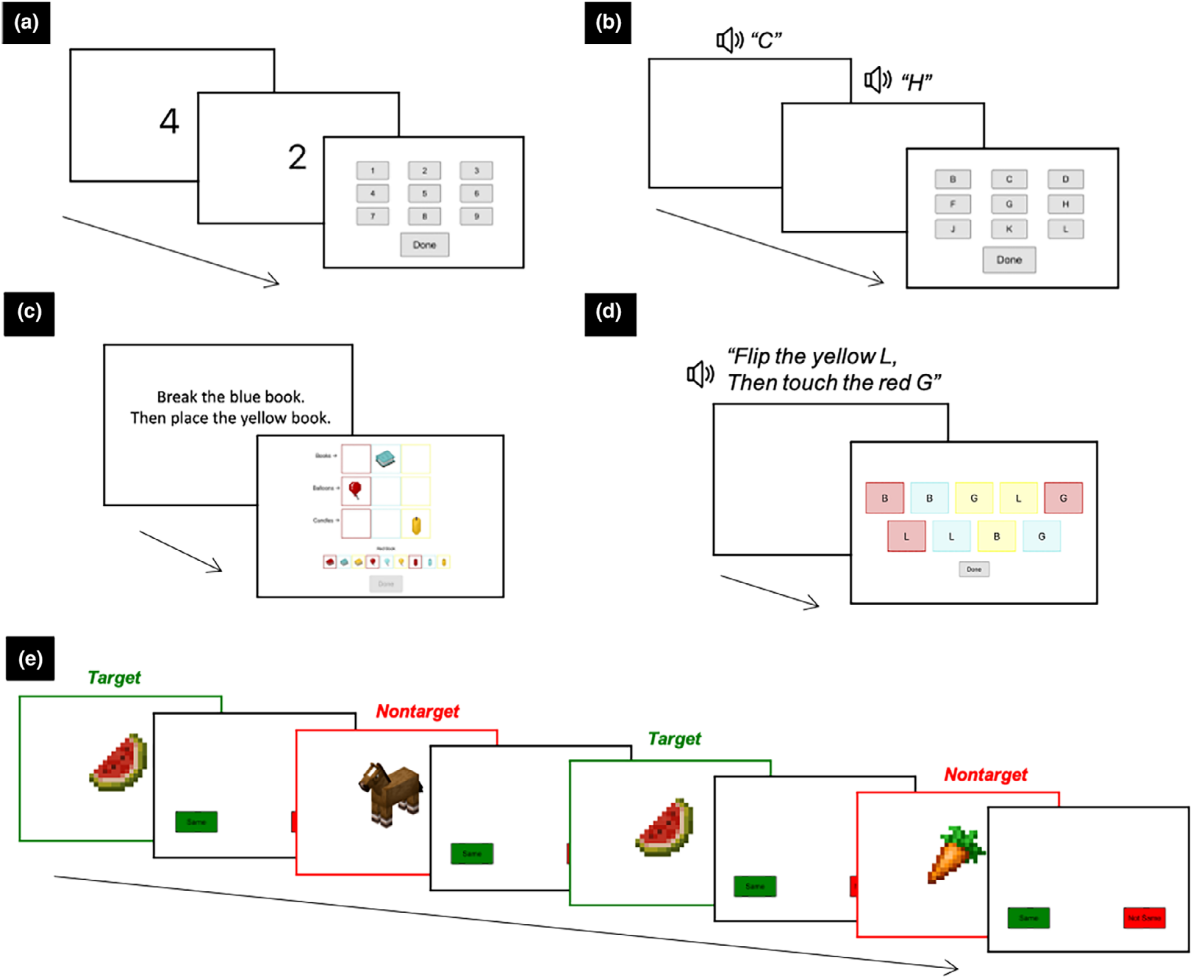
The experimental working memory transfer tests that were delivered on an iPad: (a) backward span digits, (b) backward span letters, (c) following instructions objects, (d) following instructions letters, and (e) 2‐back objects. (a–d) A span level of 2. See Lau et al. (2023) for more details.

A set of four experimental working memory tests with the same paradigms as the training activities but different stimulus features (see Lau et al., 2023) measured near transfer: (1) *backward span* tests, digits and letters versions, and (2) *following instructions* tests, objects and letters versions (see Figure 3; Gathercole et al., 2019). The set of stimuli used in the tests differed at each time point. The primary outcome measures were the backward span digits test and following instructions objects test immediately post‐intervention as they most closely resembled the training activities.

The tests, including instructions, practice items, and corrective feedback, were delivered via an iPad‐based web application developed for this research. Each test comprised blocks of four trials of a span level. Responses were self‐paced with a cut‐off duration for response time adjusted per span level. The span level increased by one if the child scored 3 or more out of the four trials in that block (≥75% accuracy); otherwise, the test ended. The total number of correct trials on each test was used as a measure of working memory performance in our analyses.

Reliability was estimated for our working memory tests by calculating internal consistency using the split‐half (odd‐even) method with Spearman‐Brown correction, consistent with procedures used for generating reliabilities of the Wechsler Intelligence Scale for Children—Fifth Edition subtests (Wechsler, 2014). For backward span tests, reliability was excellent across baseline, immediately, and 6‐months post‐intervention time points for the digits (.84, .88, and .86) and letters (.81, .86, and .82) versions. For following instructions tests, reliability was lower across time points for the objects (.65, .55, and .71) and letters (.32, .59, and .61) versions. The following instructions tests can still be considered useful for research purposes, standing out as using an ecological paradigm, even with lower reliability (Gregory, 2017).

#### Intermediate transfer

An experimental n‐back (2‐back objects) test measured intermediate transfer. This test was delivered via the same iPad‐based web application as the near transfer tests. The child was presented with a continuous stream of 22 common objects and animals (see Figure 3). The sequence of objects and animals differed at each time point. The first two trials were non‐target (not same) trials, and no response was required. For the remaining 20 trials (6 target/same and 14 non‐target/not same trials), children had to decide if the current object matched the object presented two items ago (Byrne et al., 2020; Jaeggi et al., 2010; Katz et al., 2014). The child responded by tapping the ‘same’ or ‘not same’ button within the time limit. Of the 14 non‐target/not same trials, there was one lure trial in the baseline version of the test and two lure trials in the immediate and 6‐months post‐intervention versions (for information on lure trials, see Szmalec et al., 2011).

Five n‐back scores were generated based on the signal detection theory (Peterson et al., 1954) using hits (target present and child responds correctly), correct rejections (target absent and child responds correctly), false alarms (target absent and child responds incorrectly), misses (target present and child responds incorrectly), and no responses (child did not enter a response). The signal‐detection parameter d‐prime (*d'*) was calculated and used in analyses to estimate the child's sensitivity in discriminating targets from non‐targets (Pelegrina et al., 2015). *D′* is the difference between Z‐transforms of the hit rate and the false alarm rate (*d'* = *Z*_H_ − *Z*_FA_) (Macmillan & Creelman, 1990). Reliability for the n‐back, calculated using the split‐half (odd‐even) method with Spearman‐Brown correction, was excellent across baseline, immediately, and 6‐months post‐intervention time points (.89, .87, and .85).

#### Far transfer

##### Reasoning

Children's reasoning ability was measured using the Raven's Standard Progressive Matrices (SPM), which has been standardized for children as young as 6 years through to older adults (Raven et al., 2000). The Raven's SPM contains five sets (A to E) of 12 items. Each item has a geometric design with a missing piece on the bottom right. The child has to select the correct figure from six or eight options to complete the overall geometric design vertically and horizontally. The items increase in difficulty within sets and across sets. Children were allowed as much time as they needed to complete the test. To characterize our study sample at baseline, the total number of correct items from sets A to E were converted into percentile ranges based on normative data collected in the United States (Raven et al., 2000). To determine the reasoning outcome following training, the total number of correct items from sets A and B was used in analyses (Strauss et al., 2006). The Raven's SPM has excellent spilt‐half reliability and test–retest reliability (>.80), and its concurrent validity has been well established (Raven et al., 2000). There is a moderately strong correlation (.50 to .70) between SPM and conventional intelligence tests such as Stanford‐Binet and Wechsler's tests (Strauss et al., 2006).

##### Attention

Children's inattention and hyperactivity‐impulsivity behaviors were measured using the caregiver‐rated attention‐deficit/hyperactivity disorder‐Rating Scale‐5 (ADHD‐RS‐5, home version), which is standardized for children and adolescents aged 5 to 17 years (DuPaul et al., 2016). Caregivers rated their child's behavior at home based on the past 6‐months at baseline and 6‐months post‐intervention time points and based on the past 2 weeks immediately post‐intervention to ensure no overlap in the measurement periods. The ADHD‐RS‐5 has an inattention subscale (9 items) and a hyperactivity‐impulsivity subscale (9 items), which closely follow the Diagnostic and Statistical Manual of Mental Disorders' (Fifth Edition) criteria for Attention‐Deficit/Hyperactivity Disorder. A total score was generated by summing the two subscale scores. A higher total and/or subscale score indicates more inattention and/or hyperactivity‐impulsivity behaviors. To characterize our study sample at baseline, children's total scores were converted into percentiles based on normative data collected in the United States, and percentiles at or above the 93rd percentile were used to identify children with elevated inattention and/or hyperactivity‐impulsivity behaviors (DuPaul et al., 2016). To determine inattention and hyperactivity‐impulsivity outcomes following training, the subscale scores were used in analyses. Internal consistency of the symptom subscales (.61 to .83) and concurrent validity have been established by high correlations (>.78) with respective subscales from the Connors Parent Rating Scale (DuPaul et al., 2016).

#### Child characteristics

Caregivers completed a participant information questionnaire that collected demographic information about their child, including their child's sex, developmental and medical history, and family socio‐economic risk factors (Roberts et al., 2008). Caregivers could skip questions they did not want to answer.

#### Child motivation

To account for any potential group differences in motivation that might have influenced intervention performance, children's motivation toward typical classroom activities was measured at baseline using the *Intrinsic Motivation Scale* (IMS) (Lepper et al., 2005), and their motivation toward the intervention was measured immediately post‐intervention using a modified *Intrinsic Motivation Inventory* (IMI) (Pascoe et al., 2013; Ryan & Deci, 2000a, 2000b). The IMS consists of three subscales: challenge (6 items; e.g., *I like hard work because it's difficult*), curiosity (6 items; e.g., *I ask questions in class because I want to learn new things*), and independent mastery (5 items; e.g., *I like to try to figure things out at school on my own*). The modified IMI consisted of four subscales: interest/enjoyment (7 items; e.g., *It was fun to do*), perceived competence (6 items; e.g., *I think I am pretty good at the training*), effort/importance (5 items; e.g., *I put a lot of effort into it*), and value/usefulness (4 items; e.g., *I think the training could help me*). On both questionnaires, children's responses were on a 3‐point Likert scale (yes—3 points; sometimes—2 points; no—1 point). Both questionnaires have high internal consistency (>.82) (Lepper et al., 2005; Monteiro et al., 2015). Higher scores indicate higher levels of motivation toward classroom activities or the intervention. Total raw scores from each subscale of the IMS and IMI were used to measure motivation.

### Design and procedure

This was a blinded, active‐controlled, parallel‐group, randomized superiority trial. Researchers involved in child testing were blinded to children's intervention allocation and past test results. Teachers, caregivers, and children were informed that the research study examined children's thinking skills and were unaware of the different conditions. Teachers, caregivers, and children were unaware that the study was a randomized controlled trial, thereby preserving blinding.

Assessments occurred at baseline, immediately post‐intervention (week following final intervention session), and 6‐months post‐intervention (26 weeks following final intervention session). Following baseline testing, children were randomized to one of four intervention conditions: adaptive, self‐select, stepwise working memory training, or active control. Randomization was stratified by age at baseline: 7 to 8, 9 to 10, and 11 years. However, due to a technical error, nine children from the 9 to 10 years stratification group and two children from the 11 years stratification group who were allocated to the self‐select condition performed the adaptive condition. This was discovered following completion of data collection at immediate post‐intervention (primary end point) and was due to an error made by a team member (not involved in data collection) during the intervention period for Grade 5 classes only. Therefore, the impact of this error on the trial's internal validity is expected to be negligible.

Children were tested in small groups of 6–8 children in a quiet room at the school, led by a training psychologist. The test order and administration protocol were identical at baseline, immediately post‐intervention, and at 6‐months post‐intervention. Caregivers were emailed questionnaires to complete at each time point.

### Data analyses

Analyses were carried out using R version 4.2.0 (R Core Team, 2022) implemented in Rstudio version 2022.7.2.576 (RStudio Team, 2022). Primary analyses were performed on an intention‐to‐treat basis, with sensitivity analyses only including children who completed all 10 sessions of the allocated intervention. Primary analyses were separate linear regressions for each primary outcome (backward span digits and following instructions object tests) at the primary time point (immediately post‐intervention), which were the dependent variables; the outcome measure at baseline and the stratification factor (age groups: 7–8, 9–10, 11 years; dummy coded with the 7 to 8 years age group as the reference) were covariates; and conditions (dummy coded with the active control as the reference) were independent variables. Secondary analyses were separate linear regressions for each secondary outcome (backward span letters, following instructions letters, 2‐back objects, Raven's SPM sets A and B, and ADHD‐RS‐5 caregiver rated inattention and hyperactivity). The same analytic approach was applied to secondary outcomes and both primary and secondary outcomes at the 6‐months post‐intervention time point.

Exploratory ancillary analyses were separate linear regressions for each outcome and time point that adjusted for the outcome measure at baseline, the stratification factor, and other factors that may have affected intervention outcomes, including any group differences in IMS or IMI (motivation for classroom activities or the intervention) and the differential effects of condition for child sex or age. To determine any group differences in IMS and IMI ratings, one‐way ANOVAs were conducted with each IMS and IMI subscale entered as the dependent variable and condition as the independent variable. Differential effects of condition for child sex and stratified age (7 to 8, 9 to 10, 11 years) were examined by adding the interaction term (condition × sex; condition × stratified age) to each model separately.

Statistical significance was set at *α* = .05, two‐tailed. Post hoc analyses employed Tukey's honestly significant difference test to adjust for multiple comparisons. Outcome measures were approximately normally distributed. Effect sizes (i.e., Cohen's *f* ^2^: small = 0.02, medium = 0.15, large = 0.35; Cohen's *d*: small = 0.20, medium = 0.50, large = 0.8; *η* ^2^: small = 0.01, medium = 0.06, large = 0.14) (Cohen, 1988) and 95% confidence intervals were reported to demonstrate the magnitude of differences in outcomes between each training condition relative to the active control.

Inverse Bayes factor (BF_10_) was used to quantify the strength of evidence in favor of the alternative hypothesis (working memory training condition has an effect) compared to the null (no effect of working memory training condition). BF_10_ values greater than 1 indicate increasing evidence for the alternative hypothesis (anecdotal evidence: 1–3; substantial evidence: 3–10; strong evidence: 10–30; very strong evidence: 30–100; decisive evidence: >100), while values less than 1 indicate evidence in favor of the null hypothesis (anecdotal evidence: 0.33–1.0; substantial evidence: 0.10–0.33; strong evidence: 0.03–0.10; very strong evidence: 0.01–0.03; decisive evidence: <0.01) (Jeffreys, 1961). Interpretation of results considered strength of effects as well as statistical significance.

The final data set contained 8.96% of data missing across conditions and time points. Missingness was unrelated to age, condition, sex, or outcome values at baseline. This suggests good retention and a low threat to the study's validity (>20%; Schulz & Grimes, 2002). Owing to the low proportion of missing data and the low likelihood that missingness (e.g., child was absent on day of testing) was related to outcomes of interest, listwise deletion was used.

## RESULTS

### Sample characteristics

Table 3 provides the mean and SD for each outcome measure at baseline, immediately post‐intervention, and 6‐months post‐intervention. At baseline, the intervention conditions performed similarly on the near, intermediate, and far transfer measures. For the following instructions object test, the adaptive condition had a higher mean score than the self‐select (*M*_Diff_ = 1.55, 95% CI [0.35 to 2.75], *p* = .005) and stepwise conditions (*M*_Diff_ = 1.49, 95% CI [0.41 to 2.57], *p* = .003) after adjusting for multiple comparisons. When age was controlled for, only the difference between the adaptive and stepwise conditions remained significant (*M*_Diff_ = 1.26, 95% CI [0.24 to 2.28], adjusted *p* = .008).

**TABLE 3:** Group means (*M*) and standard deviations (SD) for each outcome measure at all time points by condition for all children assessed.

	Baseline				Immediately post‐intervention				6‐Months post‐intervention			
	Active control	Adaptive training	Self‐select training	Stepwise training	Active control	Adaptive training	Self‐select training	Stepwise training	Active control	Adaptive training	Self‐select training	Stepwise training
	*M* (SD)	*M* (SD)	*M* (SD)	*M* (SD)	*M* (SD)	*M* (SD)	*M* (SD)	*M* (SD)	*M* (SD)	*M* (SD)	*M* (SD)	*M* (SD)
Near transfer												
BS digits	6.52 (3.16)	7.83 (2.98)	6.38 (2.90)	6.65 (3.12)	6.60 (3.47)	7.47 (3.98)	6.56 (3.38)	6.90 (4.05)	8.14 (3.69)	8.72 (4.51)	7.36 (3.26)	7.66 (3.51)
FI objects	6.04 (2.22)	7.08 (2.10)	5.53 (2.20)	5.59 (2.37)	6.04 (2.18)	6.91 (2.49)	6.44 (2.31)	6.46 (2.21)	7.08 (2.32)	7.65 (2.90)	7.08 (2.22)	6.90 (2.89)
BS letters	5.22 (2.20)	5.75 (2.38)	4.86 (2.34)	5.06 (2.56)	5.92 (2.57)	6.42 (3.12)	5.36 (2.36)	6.04 (3.10)	6.12 (2.46)	6.63 (2.76)	6.64 (1.82)	6.00 (3.30)
FI letters	5.82 (2.03)	6.42 (1.54)	5.92 (1.62)	6.08 (1.89)	5.94 (2.13)	6.20 (2.09)	5.58 (2.08)	5.70 (2.13)	5.98 (1.76)	6.47 (2.38)	6.19 (1.60)	5.46 (2.04)
Intermediate transfer												
2‐back objects												
Hits	3.08 (1.68)	3.43 (1.59)	3.05 (1.81)	3.02 (1.77)	3.55 (1.86)	3.93 (1.53)	3.36 (1.88)	3.68 (1.92)	4.14 (1.35)	4.35 (1.42)	4.03 (1.65)	4.12 (1.71)
CR	7.66 (3.97)	9.37 (3.91)	7.59 (3.63)	8.12 (4.27)	9.21 (3.88)	10.56 (3.06)	9.36 (3.47)	9.10 (3.62)	10.18 (3.38)	11.57 (2.65)	10.50 (3.01)	10.00 (3.06)
FA	2.08 (1.76)	1.40 (1.61)	1.97 (1.94)	1.67 (1.37)	1.38 (1.28)	1.49 (1.42)	1.58 (1.63)	1.48 (1.42)	1.47 (1.32)	1.28 (1.15)	1.31 (1.17)	1.68 (1.46)
Misses	1.06 (1.11)	1.27 (1.27)	0.86 (1.00)	0.86 (1.00)	1.02 (1.05)	1.32 (1.04)	1.36 (1.33)	0.90 (1.18)	0.90 (0.94)	1.10 (1.02)	1.11 (0.95)	0.94 (0.93)
NR^e^	6.12 (6.09)	4.54 (5.71)	6.51 (6.13)	6.33 (6.06)	4.83 (5.54)	2.70 (4.18)	4.33 (4.74)	4.84 (5.39)	3.31 (4.61)	1.70 (3.35)	3.06 (4.25)	3.26 (4.53)
d‐prime, *d′*	1.30 (0.92)	1.67 (0.94)	1.43 (0.80)	1.46 (0.86)	1.63 (0.95)	1.71 (0.84)	1.50 (0.96)	1.76 (0.84)	1.89 (0.70)	1.93 (0.83)	1.82 (0.78)	1.78 (0.76)
Far transfer												
Reasoning^a^	18.22 (3.59)	19.21 (2.95)	18.41 (3.25)	18.78 (3.64)	18.58 (3.95)	20.00 (2.61)	19.22 (3.02)	19.56 (3.20)	19.24 (3.46)	20.17 (2.55)	20.22 (1.87)	19.26 (2.83)
Inattention^b^	6.38 (6.41)	4.95 (5.35)	6.10 (4.37)	4.31 (4.65)	6.67 (6.06)	5.34 (5.16)	5.41 (3.76)	4.59 (4.32)	6.68 (6.08)	4.48 (5.00)	5.00 (4.38)	3.96 (3.87)
Hyperactivity^b^	4.38 (4.66)	3.86 (3.61)	5.03 (4.87)	2.81 (2.66)	3.94 (4.05)	2.59 (2.69)	4.77 (4.44)	2.78 (3.56)	4.86 (4.73)	2.89 (2.79)	4.27 (5.36)	2.46 (2.70)

*Note*: See Table S1 for sample sizes for each outcome measure per time point.

Abbreviations: BS, backward span; CR, correct rejections; FI, following instructions; FA, false alarms; NR, no responses.

^a^ Raven's Standard Progressive Matrices sets A and B score.

^b^ Attention‐deficit/hyperactivity disorder‐Rating Scale‐5 score.

### Child motivation

For the IMS, at baseline there were no significant differences between conditions for the challenge, *F*(3, 197) = 1.31, *p* = .271, *η* ^2^ = 0.02, or independent mastery subscales, *F*(3, 197) = 0.64, *p* = .590, *η* ^2^ = 0.01. Although there was a significant difference between conditions for the curiosity subscale *F*(3, 197) = 3.00, *p* = .032, *η* ^2^ = 0.04, post hoc analyses did not reveal any significant pairs after correcting for multiple comparisons, and the effects were small.

For the IMI, immediately post‐intervention, there were no significant differences between conditions for the perceived competence, *F*(3, 185) = 0.73, *p* = .535, *η* ^2^ = 0.01, effort/importance, *F*(3, 185) = 1.01, *p* = .388, *η* ^2^ = 0.02, or value/usefulness subscales, *F*(3, 185) = 1.61, *p* = .189, *η* ^2^ = 0.03. There was a significant difference between conditions for the interest/enjoyment subscale, *F*(3, 185) = 3.10, *p* = .028, *η* ^2^ = 0.05, with a higher mean score for the self‐select condition than the active control condition, *M*_Diff_ = 1.74, 95% CI [3.26 to 0.23], *p* = .017, *d* = 0.63.

Examination of the potential effect of age or child sex on children's motivation toward classroom activities and the intervention revealed that males (*M =* 14.50, SD = 2.22) rated themselves as having lesser curiosity toward classroom activities than females (*M* = 15.51, SD = 1.96), *t*(199) = −3.40, *p* < .001, *d* = 0.48. Males (*M* = 12.86, SD = 2.11), and rated themselves as having higher effort/importance toward the intervention than females (*M* = 13.47, SD = 1.53), *t*(187) = −2.28, *p* = .024, *d* = 0.33. All other comparisons did not reach statistical significance and had negligible to small effect sizes (*d* = 0.01 to 0.22).

### Outcomes immediately post‐intervention

Table 4 summarizes the results at immediately post‐intervention. None of the working memory training conditions performed significantly different from the active control on the outcome measures, with effect sizes ranging from negligible to small (*f* ^2^ = 0.00 to 0.04). Sensitivity analyses including only children who completed all 10 intervention sessions showed similar results (*p* = .24 to .96, *f* ^2^ = 0.00 to 0.05). Children's interest/enjoyment of the intervention at the immediately post‐intervention time point was not a significant predictor of any outcome score. Results of Bayesian analyses revealed strong to very strong (BF_10_ = 0.03 to 0.09) evidence for the null hypothesis for most outcome measures immediately post‐intervention, with the exception of inattention and hyperactivity (BF_10_ = 0.19 to 0.25, substantial evidence for the null hypothesis), suggesting the different methods used to adjust the difficulty of the training activities did not impact near, intermediate, or far transfer outcomes.

**TABLE 4:** Comparisons from baseline to immediately post‐intervention, and baseline to 6‐months post‐intervention.

Transfer range/parameter	Immediately post‐intervention				6‐Months post‐intervention			
	Est. [95% CI]	*p*	*f* ^2^	BF_10_	Est. [95% CI]	*p*	*f* ^2^	BF_10_
Near transfer—backward span digits								
Intercept	2.07 [0.73, 3.41]	.003			4.55 [3.07, 6.02]	<.001		
Baseline scores	0.59 [0.43, 0.74]	<.001	0.31		0.43 [0.26, 0.60]	<.001	0.14	
Stratified age groups			0.07				0.05	
9–10 years versus 7–8 years	1.41 [0.46, 2.36]	.004			1.44 [0.38, 2.49]	.008		
11 years versus 7–8 years	2.87 [0.75, 5.00]	.008			2.24 [0.05, 4.43]	.045		
Condition			0.00	0.03			0.00	0.04
Adaptive versus active control	−0.18 [−1.42, 1.06]	.770			−0.22 [−1.57, 1.12]	.743		
Self‐select versus active control	0.21 [−1.15, 1.58]	.759			−0.51 [−2.03, 1.00]	.505		
Stepwise versus active control	0.10 [−1.15, 1.35]	.875			−0.50 [−1.89, 0.88]	.473		
Near transfer—following instructions objects								
Intercept	3.51 [2.52, 4.50]	<.001			4.37 [3.21, 5.53]	<.001		
Baseline scores	0.31 [0.17, 0.45]	<.001	0.11		0.35 [0.19, 0.51]	<.001	0.09	
Stratified age groups			0.10				0.06	
9–10 years versus 7–8 years	1.24 [0.61, 1.88]	<.001			1.05 [0.31, 1.78]	.005		
11 years versus 7–8 years	1.95 [0.53, 3.37]	.008			1.95 [0.42, 3.47]	.013		
Condition			0.02	0.09			0.01	0.05
Adaptive versus active control	0.31 [−0.50, 1.11]	.453			−0.01 [−0.93, 0.91]	.980		
Self‐select versus active control	0.73 [−0.16, 1.63]	.108			0.50 [−0.54, 1.54]	.345		
Stepwise versus active control	0.49 [−0.33, 1.31]	.236			0.00 [−0.94, 0.95]	.997		
Near transfer—backward span letters								
Intercept	2.69 [1.67, 3.71]	<.001			4.04 [2.98, 5.10]	<.001		
Baseline scores	0.51 [0.36, 0.66]	<.001	0.24		0.32 [0.17, 0.48]	<.001	0.09	
Stratified age groups			0.10				0.06	
9–10 years versus 7–8 years	1.07 [0.33, 1.81]	.005			0.58 [−0.19, 1.35]	.141		
11 years versus 7–8 years	3.12 [1.50, 4.73]	<.001			2.67 [1.11, 4.23]	.001		
Condition			0.00	0.03			0.02	0.04
Adaptive versus active control	−0.05 [−0.99, 0.88]	.914			0.12 [−0.83, 1.07]	.804		
Self‐select versus active control	−0.23 [−1.27, 0.81]	.659			0.78 [−0.30, 1.86]	.155		
Stepwise versus active control	0.08 [−0.88, 1.03]	.876			−0.06 [−1.05, 0.92]	.898		
Near transfer—following instructions letters								
Intercept	3.37 [2.25, 4.49]	<.001			4.07 [2.97, 5.16]	<.001		
Baseline scores	0.36 [0.20, 0.53]	<.001	0.10		0.26 [0.10, 0.42]	.002	0.05	
Stratified age groups			0.05				0.04	
9–10 years versus 7–8 years	0.78 [0.19, 1.37]	.010			0.79 [0.21, 1.36]	.008		
11 years versus 7–8 years	1.29 [−0.04, 2.62]	.057			0.72 [−0.48, 1.93]	.239		
Condition			0.00	0.04			0.03	0.28
Adaptive versus active control	−0.11 [−0.89, 0.68]	.787			0.20 [−0.55, 0.96]	.597		
Self‐select versus active control	−0.25 [−1.11, 0.61]	.571			0.28 [−0.57, 1.12]	.515		
Stepwise versus active control	−0.33 [−1.12, 0.46]	.415			−0.56 [−1.34, 0.21]	.151		
Intermediate transfer—2‐back objects								
Intercept	1.14 [0.83, 1.44]	<.001			1.49 [1.22, 1.76]	<.001		
Baseline scores	0.19 [0.05, 0.33]	.007	0.04		0.19 [0.07, 0.32]	.003	0.05	
Stratified age groups			0.09				0.03	
9–10 years versus 7–8 years	0.51 [0.26, 0.76]	<.001			0.27 [0.05, 0.50]	.017		
11 years versus 7–8 years	0.53 [−0.04, 1.10]	.070			0.24 [−0.23, 0.72]	.313		
Condition			0.01	0.05			0.00	0.04
Adaptive versus active control	−0.06 [−0.39, 0.26]	.700			−0.06 [−0.34, 0.23]	.697		
Self‐select versus active control	−0.13 [−0.49, 0.23]	.486			−0.06 [−0.38, 0.27]	.722		
Stepwise versus active control	0.07 [−0.26, 0.40]	.684			−0.12 [−0.42, 0.17]	.408		
Far transfer—reasoning^a^								
Intercept	5.31 [3.52, 7.11]	<.001			9.45 [7.52, 11.39]	<.001		
Baseline scores	0.73 [0.63, 0.83]	<.001	1.14		0.55 [0.44, 0.65]	<.001	0.54	
Stratified age groups			0.00				0.01	
9–10 years versus 7–8 years	0.03 [−0.65, 0.70]	.938			−0.49 [−1.19, 0.22]	.177		
11 years versus 7–8 years	0.32 [−1.13, 1.77]	.662			0.22 [−1.19, 1.63]	.759		
Condition			0.01	0.07			0.03	0.22
Adaptive versus active control	0.61 [−0.21, 1.43]	.144			0.35 [−0.49, 1.20]	.414		
Self‐select versus active control	0.49 [−0.42, 1.40]	.291			0.82 [−0.14, 1.78]	.092		
Stepwise versus active control	0.37 [−0.47, 1.21]	.386			−0.21 [−1.08, 0.67]	.640		
Far transfer—inattention^b^								
Intercept	2.51 [1.38, 3.63]	<.001			1.42 [−0.01, 2.85]	.051		
Baseline scores	0.76 [0.67, 0.84]	<.001	3.20		0.73 [0.63, 0.82]	<.001	3.04	
Stratified age groups			0.05				0.03	
9–10 years versus 7–8 years	−1.04 [−1.98, −0.09]	.031			−0.50 [−1.62, 0.62]	.379		
11 years versus 7–8 years	−1.25 [−3.01, 0.50]	.160			−1.40 [−3.37, 0.56]	.158		
Condition			0.03	0.19			0.01	0.08
Adaptive versus active control	−0.48 [−1.72, 0.76]	.446			−0.17 [−1.66, 1.32]	.819		
Self‐select versus active control	−1.13 [−2.48, 0.22]	.101			−0.62 [−2.30, 1.06]	.465		
Stepwise versus active control	−0.92 [−2.14, 0.31]	.140			0.00 [−1.51, 1.51]	.999		
Far transfer—hyperactivity^b^								
Intercept	0.97 [−0.08, 2.01]	.071			0.74 [−0.54, 2.03]	.253		
Baseline scores	0.75 [0.64, 0.86]	<.001	1.79		0.77 [0.65, 0.89]	<.001	2.09	
Stratified age groups			0.01				0.02	
9–10 years versus 7–8 years	−0.33 [−1.21, 0.56]	.465			0.42 [−0.61, 1.46]	.419		
11 years versus 7–8 years	−0.58 [−2.23, 1.07]	.486			−0.54 [−2.31, 1.24]	.550		
Condition			0.04	0.25			0.03	0.16
Adaptive versus active control	−0.99 [−2.14, 0.16]	.090			−0.79 [−2.13, 0.54]	.240		
Self‐select versus active control	0.12 [−1.13, 1.37]	.854			−1.07 [−2.58, 0.44]	.163		
Stepwise versus active control	−0.18 [−1.31, 0.95]	.755			−0.51 [−1.87, 0.84]	.455		

*Note*: See Table S2 for omnibus test results and variance explained.

Abbreviation: Est., estimate.

^a^ Raven's Standard Progressive Matrices sets A and B score.

^b^ Attention‐deficit/hyperactivity disorder‐Rating Scale‐5 score.

### Outcomes 6‐months post‐intervention

Table 4 summarizes the results at 6‐months post‐intervention. None of the working memory training conditions performed significantly different from the active control on the outcome measures, with effect sizes ranging from negligible to small (*f* ^2^ = 0.00 to 0.03). Sensitivity analyses including only children who completed all 10 intervention sessions showed similar results (*p* = .16 to .99, *f* ^2^ = 0.00 to 0.04). Children's interest/enjoyment of the intervention immediately post‐intervention was not a significant predictor of any outcome score at 6‐months post‐intervention. Results of Bayesian analyses revealed substantial to very strong (BF_10_ = 0.04 to 0.28) evidence for the null hypothesis for outcome measures at 6‐months post‐intervention, suggesting the different methods used to adjust the difficulty of the training activities did not impact near, intermediate, or far transfer outcomes.

### Exploratory ancillary analyses

#### Controlling for child motivation

Baseline curiosity (IMS) and post‐intervention interest/enjoyment (IMI) were not associated with outcomes between working memory training conditions and the active control at immediately or at 6‐months post‐intervention (see Table S3). The effect sizes ranged from negligible to small (*f* ^2^ = 0.00 to 0.03).

#### Differential effects of child sex

There were generally no differences in outcomes between the working memory training conditions and the active control for males and females immediately post‐intervention and 6‐months post‐intervention (see Table S4). The exception was the hyperactivity measure immediately post‐intervention, with post hoc comparisons revealing that males in the active control (*n* = 31, estimated marginal mean = 3.98, 95% CI [2.89, 5.07]) were rated more hyperactive than males in the adaptive condition (*n* = 34, estimated marginal mean = 1.73, 95% CI [0.57, 2.88]), *p* = .004, Cohen's *d* = 1.05.

#### Differential effects of child age

There were generally no differences in outcomes between the working memory training conditions and the active control for children aged 7 to 8 and 11 years immediately post‐intervention, with the exception of the backward span letters test (see Table S5). Post hoc analyses revealed that 9‐ to 10‐year‐olds in the adaptive condition (*n* = 33, estimated marginal mean = 7.13, 95% CI [6.28, 7.97]) performed better than those in the self‐select condition (*n* = 16, estimated marginal mean = 5.06, 95% CI [3.88, 6.23]), *p* = .005, Cohen's *d* = 0.42. This result should be interpreted with caution due to the difference in sample size between groups. There were no differences in outcomes at 6‐months post‐intervention between the working memory training conditions and the active control for children of different ages.

## DISCUSSION

Despite the near‐universal adoption of an adaptive approach for setting the difficulty of activities in cognitive training programs, only one previous study (von Bastian & Eschen, 2016) has systematically compared performances in adults using adaptive and non‐adaptive approaches. The current study extends this work by comparing in children adaptive and non‐adaptive methods of working memory training to an active control on a range of outcome measures. Children in the adaptive, self‐select, and stepwise working memory training conditions performed similarly on near, intermediate, and far transfer measures immediately and 6‐months post‐intervention.

Our findings in children are remarkably consistent with those of von Bastian and Eschen (2016). In that study, difficulty of working memory training activities were adjusted using adaptive, self‐select, or randomized approaches and compared outcomes to an active control. While near transfer was not measured, the adults in their adaptive condition performed similarly to those in the self‐select condition, randomized condition, and active control on intermediate and far transfer measures immediately post‐intervention. Together with this previous study, our study results challenge the assumption that an adaptive approach maximizes cognitive training outcomes and suggest that varying training difficulty through other non‐adaptive approaches (i.e., self‐select, stepwise, and randomized) can produce similar training (null) outcomes.

While the lack of improvements we observed following working memory training on intermediate and far transfer measures is consistent with results of high‐quality randomized control trials (Roberts et al., 2016; von Bastian & Eschen, 2016) and meta‐analyses (Kassai et al., 2019; Sala & Gobet, 2020), the lack of near‐transfer effects is not. We speculate this might be attributable to at least two factors. Firstly, very few children had low reasoning skills (i.e., below the 25th percentile) or had caregiver‐reported diagnoses (i.e., ADHD, autism spectrum disorder, anxiety, learning difficulties), elevated hyperactivity, or inattention. Thus, our sample of children from a mainstream primary school may be performing within age expectations, differentiating them from a selected clinical sample where working memory might be low and thus, perhaps, a greater opportunity for improvement. Similarly, in a sample of mostly average‐ability children aged 10 to 13 years, Hitchcock and Westwell (2017) also found no evidence of near transfer following working memory training (*n* = 54) compared with non‐adaptive training (*n* = 45) or a passive control (*n* = 49) condition. Our findings, like those of Hitchcock and Westwell (2017), may not be generalized to populations experiencing difficulties. Indeed, some small sample working memory training studies in clinical samples, such as those with ADHD (Holmes et al., 2009, 2010; Klingberg et al., 2002, 2005) or with lower abilities in the cognitive skill trained (Jaeggi et al., 2008), have reported transfer (von Bastian & Oberauer, 2014). We acknowledge, however, that not all trials of working memory training in selected child samples have observed transfer, for example, in children born extremely preterm (Anderson et al., 2018) and children screened for low working memory (Roberts et al., 2016). Second, while aspects of the intervention design were informed by current evidence of working memory training methods, such as including a backward span training activity—chosen for its demonstrated higher transfer effects compared to other working memory training paradigms like serial recall (recalling items in the order they were presented), complex span (serial recall is interleaved with a secondary distractor task) and n‐back (Gathercole et al., 2019; Soveri et al., 2017)—other untested design features, such as training dose and number of training activities, might have contributed to the lack of near transfer effects we observed. It is interesting to note that in children with low working memory, Roberts et al. (2016) observed improvements on two of their four working memory outcome measures at 6‐months post‐intervention after a higher dose of training (i.e., 5 times a week for 5 weeks, total training dose: 600–1500 min).

In our study, children in the working memory training conditions rated similar levels of motivation (interest/enjoyment, perceived competence, effort/importance, value/usefulness) toward the intervention. This represents a first attempt to explore children's motivation toward adaptive and non‐adaptive approaches of working memory training. The only previous study to explore this was in adults, where von Bastian and Eschen (2016) also observed comparable motivation levels toward adaptive and self‐select approaches of working memory training. Our finding is of considerable practical value in showing that in younger populations, non‐adaptive approaches to setting difficulty of training activities are no less motivating than adaptive approaches. While important concerns have been raised about the use of responder analysis to determine whether working memory training leads to transfer (Tidwell et al., 2014), it is noted that Jaeggi et al. (2011) identified motivational differences among children in their adaptive working memory training group (*n* = 32). A subgroup of children who showed no improvements following training rated the training as more difficult and effortful (*n* = 16), while a second subgroup that displayed improvements on outcome measures rated it as challenging but not overwhelming (*n* = 16). They postulated that the adaptive difficulty increments used in their training might have been too large for some children (i.e., beyond their zone of proximal development) and suggested implementing more trials on a given level to support opportunities to master the learning (i.e., scaffolding). Our training conditions included several design features that scaffolded children and aimed to ensure children were within their zone of proximal development when training, which might explain why children's motivation toward the different training conditions (especially the preset adaptive and stepwise approaches) were similar despite the distinct approaches to adjusting difficulty levels. Specifically: (1) starting the training on an activity at the lowest span for all three approaches, (2) setting the threshold for adjusting the difficulty downward (i.e., two incorrect trials) lower than the threshold for adjusting the difficulty upward (i.e., three correct trials) in the adaptive approach, (3) adapting training difficulty for the first four blocks and maintaining the same span from the fourth to fifth (last) block to allow for more practice at the last span achieved at the end of the session for the adaptive approach, (4) starting each adaptive training session with the span level attained at the end of the previous session minus one, (5) restricting difficulty adjustments in the self‐select approach to one span (increase or decrease by one level) per session, (6) tailoring the stepwise progression to children's working memory development (Gathercole et al., 2004), and (7) applying session‐by‐session difficulty adjustment in the self‐select and stepwise approaches to enable extended practice at a given span level.

We acknowledge that in our exploratory analyses, there were select differential effects of sex or age observed in the adaptive working memory training condition compared with the control immediately post‐intervention. Males in the adaptive condition, compared with the active control, had significantly lower caregiver‐rated hyperactive scores. It is challenging to understand the potential cognitive mechanisms leading to improvements in males' hyperactive behaviors, given we did not observe a similar pattern of improvements for other working memory training conditions or improvements on nearer transfer tests (Pahor et al., 2022; von Bastian et al., 2022).

Strengths of the current study include a rigorous experimental design and the adoption of best practices in cognitive training studies, thus providing confidence in the findings (Gobet & Sala, 2023; Simons et al., 2016). Importantly, the working memory training conditions and active control were designed to be similar on key design aspects (i.e., Minecraft environment, motivational factors, social contact) so that the active control could be considered a placebo and ensure blinding. We selected creative building activities that do not load on children's working memory. Including baseline motivation and motivation toward the intervention in sensitivity analyses further accounted for potential extraneous effects (Parong et al., 2022). Eight transfer outcome measures were used to detect discrete transfer levels, including four near‐transfer tests, addressing limitations and minimizing potential errors associated with using a single measure. Our sample size of 37 to 63 children per condition (*N* = 201) was sufficiently powered (>80% power) to address the study aim and primary analyses. Minecraft is popular with children and may have been a factor in obtaining high levels of caregiver consent (94%), intervention completion across the four conditions (70% to 82%), and retention at 6‐months post‐intervention (97%). Finally, our statistical methods involved correction for multiple tests to minimize false positives and Bayesian analysis techniques to quantify the evidence for the alternative hypothesis that training had a genuine effect compared to the null.

A limitation of our study is that we did not address other design features of working memory training that may influence training effects and transfer. These design features include training dose, diversity of training activities, and scheduling of the intervention sessions. For example, children could complete up to three training sessions within a single day during the intervention period to maximize the opportunity to complete all 10 training sessions, but this decision might have impacted the effect of spacing in learning, and thus the findings. The active control was not designed to target working memory, and including a no‐contact control (teaching as usual) would have confirmed whether it unintentionally involved working memory or not. We chose not to include a no‐contact control condition due to concerns around blinding integrity; for example, even if randomization was at the class (rather than child) level, conversation about the training program between children and teachers across classes could have resulted in contamination. It is possible that the repeated administration of the Raven's SPM Sets A and B at baseline, immediately, and 6‐months post‐intervention reduced the novelty of the items and potentially impacted the measure's ability to detect differences in reasoning outcomes between conditions. The technical error that resulted in some 10‐ to 11‐year‐olds performing the adaptive instead of the self‐select condition was another limitation that may have affected exploratory analyses of child age.

Future research into other design features of working memory training in children might provide insight into our findings. There are many methods to adapt the difficulty level of training activities to the individual's performance, and the present study examined one such algorithm where training difficulty was adjusted on a block‐by‐block basis. Other adaptive algorithms that have been used include adjusting the difficulty level on a trial‐by‐trial basis (Cogmed; Klingberg et al., 2005) or on a session‐by‐session basis (Lumosity; Steyvers & Schafer, 2020). Equally rigorous trials could explore the differential effects of other adaptive algorithms and evaluate the adaptive approach in cognitive training targeting other domains.

## CONCLUSION

In children, the adaptive approach to adjusting the difficulty of working memory training activities was not superior to the studied non‐adaptive approaches when compared with the active control. Thus, our results question the widely held assumption that the adaptive approach is a *principle* of working memory training. No effects of working memory training were observed. It is important to consider the child population studied and transfer measures used when making conclusions across studies, which might have different aims, methodologies, and outcomes of interest (Green et al., 2019).

## AUTHOR CONTRIBUTIONS

Regine C. Lau: conceptualization, data curation, formal analysis, funding acquisition, investigation, methodology, project administration, resources, visualization, writing—original draft, writing—review & editing; Peter J. Anderson: conceptualization, methodology, supervision, writing—review & editing; Susan Gathercole: conceptualization; methodology; writing—review & editing; Joshua F. Wiley: data curation; formal analysis; methodology; supervision; writing—review & editing; Megan M. Spencer‐Smith: conceptualization; funding acquisition; methodology; supervision; writing—review & editing; project administration.

## CONFLICT OF INTEREST STATEMENT

All authors declare that they have no competing interests.

## Supporting information


Data S1.

## Data Availability

The data necessary to reproduce the analyses presented here are not publicly accessible. To request and receive data, please contact the study investigators megan.spencer-smith@monash.edu, peter.j.anderson@monash.edu and joshua.wiley@monash.edu. The analytic code necessary to reproduce the analyses presented in this paper is not publicly accessible, nor are the materials necessary to attempt to replicate the findings. The trial design and analyses presented here were registered retrospectively with the Australian New Zealand Clinical Trials Registry, accessible at https://www.anzctr.org.au/ACTRN12621000990820.aspx.

## References

[cdev14180-bib-0001] Anderson, P. J. , Lee, K. J. , Roberts, G. , Spencer‐Smith, M. M. , Thompson, D. K. , Seal, M. L. , Nosarti, C. , Grehan, A. , Josev, E. K. , Gathercole, S. , Doyle, L. W. , & Pascoe, L. (2018). Long‐term academic functioning following Cogmed working memory training for children born extremely preterm: A randomized controlled trial. Journal of Pediatrics, 202, 92–97.e4. 10.1016/j.jpeds.2018.07.003 30177350

[cdev14180-bib-0002] Astle, D. E. , Barnes, J. J. , Baker, K. , Colclough, G. L. , & Woolrich, M. W. (2015). Cognitive training enhances intrinsic brain connectivity in childhood. Journal of Neuroscience, 35(16), 6277–6283. 10.1523/JNEUROSCI.4517-14.2015 25904781 PMC4405549

[cdev14180-bib-0003] Byrne, E. M. , Ewbank, M. P. , Gathercole, S. E. , & Holmes, J. (2020). The effects of transcranial direct current stimulation on within‐ and cross‐paradigm transfer following multi‐session backward recall training. Brain and Cognition, 141, 105552. 10.1016/j.bandc.2020.105552 32298870 PMC7221346

[cdev14180-bib-0004] Cohen, J. (1988). Statistical power analysis for the behavioral sciences (2nd ed.). Lawrence Erlbaum Associates.

[cdev14180-bib-0005] Dunning, D. L. , Holmes, J. , & Gathercole, S. E. (2013). Does working memory training lead to generalized improvements in children with low working memory? A randomized controlled trial. Developmental Science, 16(6), 915–925. 10.1111/desc.12068 24093880 PMC4232921

[cdev14180-bib-0006] DuPaul, G. J. , Power, T. J. , Anastopoulos, A. D. , & Reid, R. (2016). ADHD Rating Scale‐5 for children and adolescents. In Checklists, norms, and clinical interpretation (Vol. 5). Guilford Publications.

[cdev14180-bib-0007] Erdfelder, E. , FAul, F. , Buchner, A. , & Lang, A. G. (2009). Statistical power analyses using G*Power 3.1: Tests for correlation and regression analyses. Behavior Research Methods, 41(4), 1149–1160. 10.3758/BRM.41.4.1149 19897823

[cdev14180-bib-0008] Faul, F. , Erdfelder, E. , Lang, A. G. , & Buchner, A. (2007). G*Power 3: A flexible statistical power analysis program for the social, behavioral, and biomedical sciences. Behavior Research Methods, 39(2), 175–191. 10.3758/BF03193146 17695343

[cdev14180-bib-0009] Gathercole, S. E. , Dunning, D. L. , Holmes, J. , & Norris, D. (2019). Working memory training involves learning new skills. Journal of Memory and Language, 105, 19–42. 10.1016/j.jml.2018.10.003 PMC6591133 31235992

[cdev14180-bib-0010] Gathercole, S. E. , Durling, E. , Evans, M. , Jeffcock, S. , & Stone, S. (2008). Working memory abilities and children's performance in laboratory analogues of classroom activities. Applied Cognitive Psychology, 22(8), 1019–1037. 10.1002/acp.1407

[cdev14180-bib-0011] Gathercole, S. E. , Pickering, S. J. , Ambridge, B. , & Wearing, H. (2004). The structure of working memory from 4 to 15 years of age. Developmental Psychology, 40(2), 177–190. 10.1037/0012-1649.40.2.177 14979759

[cdev14180-bib-0012] Gobet, F. , & Sala, G. (2023). Cognitive training: A field in search of a phenomenon. Perspectives on Psychological Science, 18(1), 125–141. 10.1177/17456916221091830 35939827 PMC9903001

[cdev14180-bib-0013] Green, C. S. , Bavelier, D. , Kramer, A. F. , Vinogradov, S. , Ansorge, U. , Ball, K. K. , Bingel, U. , Chein, J. M. , Colzato, L. S. , Edwards, J. D. , Facoetti, A. , Gazzaley, A. , Gathercole, S. E. , Ghisletta, P. , Gori, S. , Granic, I. , Hillman, C. H. , Hommel, B. , Jaeggi, S. M. , … Witt, C. M. (2019). Improving methodological standards in behavioral interventions for cognitive enhancement. Journal of Cognitive Enhancement, 3(1), 2–29. 10.1007/s41465-018-0115-y

[cdev14180-bib-0014] Gregory, R. J. (2017). Concepts of Reliability. In Handbook of item response theory (7th ed.). Pearson Education, 99–117.

[cdev14180-bib-0015] Hitchcock, C. , & Westwell, M. S. (2017). A cluster‐randomised, controlled trial of the impact of Cogmed working memory training on both academic performance and regulation of social, emotional and behavioural challenges. Journal of Child Psychology and Psychiatry, and Allied Disciplines, 58(2), 140–150. 10.1111/jcpp.12638 27718248

[cdev14180-bib-0016] Holmes, J. , Gathercole, S. E. , & Dunning, D. L. (2009). Adaptive training leads to sustained enhancement of poor working memory in children. Developmental Science, 12(4), 9–15. 10.1111/j.1467-7687.2009.00848.x 19635074

[cdev14180-bib-0017] Holmes, J. , Gathercole, S. E. , Place, M. , Dunning, D. L. , Hilton, K. A. , & Elliott, J. G. (2010). Working memory deficits can be overcome: Impacts of training and medication on working memory in children with ADHD. Applied Cognitive Psychology, 24(6), 827–836. 10.1002/ACP.1589

[cdev14180-bib-0018] Jaeggi, S. M. , Buschkuehl, M. , Jonides, J. , & Perrig, W. J. (2008). Improving fluid intelligence with training on working memory. Proceedings of the National Academy of Sciences of the United States of America, 105(19), 6829–6833. 10.1073/pnas.0801268105 18443283 PMC2383929

[cdev14180-bib-0019] Jaeggi, S. M. , Buschkuehl, M. , Jonides, J. , & Shah, P. (2011). Short‐ and long‐term benefits of cognitive training. Proceedings of the National Academy of Sciences of the United States of America, 108(25), 10081–10086. 10.1073/pnas.1103228108 21670271 PMC3121868

[cdev14180-bib-0020] Jaeggi, S. M. , Buschkuehl, M. , Perrig, W. J. , & Meier, B. (2010). The concurrent validity of the N‐back task as a working memory measure. Memory, 18(4), 394–412. 10.1080/09658211003702171 20408039

[cdev14180-bib-0021] Jeffreys, H. (1961). The theory of probability (3rd ed.). Clarendon Press.

[cdev14180-bib-0022] Jones, J. S. , Adlam, A. L. R. , Benattayallah, A. , & Milton, F. N. (2022). The neural correlates of working memory training in typically developing children. Child Development, 93(3), 815–830. 10.1111/CDEV.13721 34897651

[cdev14180-bib-0023] Karbach, J. , Strobach, T. , & Schubert, T. (2015). Adaptive working‐memory training benefits reading, but not mathematics in middle childhood. Child Neuropsychology, 21(3), 285–301. 10.1080/09297049.2014.899336 24697256

[cdev14180-bib-0024] Kassai, R. , Futo, J. , Demetrovics, Z. , & Takacs, Z. K. (2019). A meta‐analysis of the experimental evidence on the near‐ and far‐transfer effects among children's executive function skills. Psychological Bulletin, 145(2), 165–188. 10.1037/bul0000180 30652908

[cdev14180-bib-0025] Katz, B. , Jaeggi, S. , Buschkuehl, M. , Stegman, A. , & Shah, P. (2014). Differential effect of motivational features on training improvements in school‐based cognitive training. Frontiers in Human Neuroscience, 8, 242. 10.3389/fnhum.2014.00242 24795603 PMC4006056

[cdev14180-bib-0026] Kelly, C. E. , Harding, R. , Lee, K. J. , Pascoe, L. , Josev, E. K. , Spencer‐Smith, M. M. , Adamson, C. , Beare, R. , Nosarti, C. , Roberts, G. , Doyle, L. W. , Seal, M. L. , Thompson, D. K. , & Anderson, P. J. (2021). Investigating the brain structural connectome following working memory training in children born extremely preterm or extremely low birth weight. Journal of Neuroscience Research, 99(10), 2340–2350. 10.1002/JNR.24818 33624327

[cdev14180-bib-0027] Kelly, C. E. , Thompson, D. K. , Chen, J. , Josev, E. K. , Pascoe, L. , Spencer‐Smith, M. M. , Adamson, C. , Nosarti, C. , Gathercole, S. , Roberts, G. , Lee, K. J. , Doyle, L. W. , Seal, M. L. , & Anderson, P. J. (2020). Working memory training and brain structure and function in extremely preterm or extremely low birth weight children. Human Brain Mapping, 41, 3–696. 10.1002/hbm.24832 PMC6977425 31713952

[cdev14180-bib-0028] Klingberg, T. (2010). Training and plasticity of working memory. Trends in Cognitive Sciences, 14(7), 317–324. 10.1016/j.tics.2010.05.002 20630350

[cdev14180-bib-0029] Klingberg, T. , Fernell, E. , Olesen, P. J. , Johnson, M. , Gustafsson, P. , Dahlström, K. , Gillberg, C. G. , Forssberg, H. , & Westerberg, H. (2005). Computerized training of working memory in children with ADHD—A randomized, controlled trial. Journal of the American Academy of Child and Adolescent Psychiatry, 44(2), 177–186. 10.1097/00004583-200502000-00010 15689731

[cdev14180-bib-0030] Klingberg, T. , Forssberg, H. , & Westerberg, H. (2002). Training of working memory in children with ADHD. Journal of Clinical and Experimental Neuropsychology, 24(6), 781–791. 10.1076/jcen.24.6.781.8395 12424652

[cdev14180-bib-0031] Lau, R. C. , Anderson, P. J. , Wiley, J. F. , Huang, D. , Surjatin, F. , McIntosh, P. , Gathercole, S. , & Spencer‐Smith, M. (2023). Working memory training for children using the adaptive, self‐select, and stepwise approaches to setting the difficulty level of training activities: Protocol for a randomized controlled trial. JMIR Research Protocols, 12(1), e47496. 10.2196/47496 37725418 PMC10548317

[cdev14180-bib-0032] Lepper, M. R. , Corpus, J. H. , & Iyengar, S. S. (2005). Intrinsic and extrinsic motivational orientations in the classroom: Age differences and academic correlates. Journal of Educational Psychology, 97(2), 184–196. 10.1037/0022-0663.97.2.184

[cdev14180-bib-0033] Loosli, S. V. , Buschkuehl, M. , Perrig, W. J. , & Jaeggi, S. M. (2012). Working memory training improves reading processes in typically developing children. Child Neuropsychology, 18(1), 62–78. 10.1080/09297049.2011.575772 21623483

[cdev14180-bib-0034] Lövdén, M. , Bäckman, L. , Lindenberger, U. , Schaefer, S. , & Schmiedek, F. (2010). A theoretical framework for the study of adult cognitive plasticity. Psychological Bulletin, 136(4), 659–676. 10.1037/a0020080 20565172

[cdev14180-bib-0035] Macmillan, N. A. , & Creelman, C. D. (1990). Response bias: Characteristics of detection theory, threshold theory, and “nonparametric” indexes. Psychological Bulletin, 107(3), 401–413. 10.1037/0033-2909.107.3.401

[cdev14180-bib-0036] Melby‐Lervåg, M. , & Hulme, C. (2013). Is working memory training effective? A meta‐analytic review. Developmental Psychology, 49(2), 270–291. 10.1037/a0028228 22612437

[cdev14180-bib-0037] Melby‐Lervåg, M. , Redick, T. S. , & Hulme, C. (2016). Working memory training does not improve performance on measures of intelligence or other measures of “far transfer”: Evidence from a meta‐analytic review. Perspectives on Psychological Science, 11(4), 512–534. 10.1177/1745691616635612 27474138 PMC4968033

[cdev14180-bib-0038] Monteiro, V. , Mata, L. , & Peixoto, F. (2015). Intrinsic Motivation Inventory: Psychometric properties in the context of first language and mathematics learning. Psicologia: Reflexao e Critica, 28(3), 434–443. 10.1590/1678-7153.201528302

[cdev14180-bib-0039] Pahor, A. , Seitz, A. R. , & Jaeggi, S. M. (2022). Near transfer to an unrelated N‐back task mediates the effect of N‐back working memory training on matrix reasoning. Nature Human Behaviour, 6(9), 1243–1256. 10.1038/s41562-022-01384-w PMC12305750 35726054

[cdev14180-bib-0040] Parong, J. , Seitz, A. R. , Jaeggi, S. M. , & Green, C. S. (2022). Expectation effects in working memory training. Proceedings of the National Academy of Sciences of the United States of America, 119(37), e2209308119. 10.1073/pnas.2209308119 36067292 PMC9477404

[cdev14180-bib-0041] Pascoe, L. , Roberts, G. , Doyle, L. W. , Lee, K. J. , Thompson, D. K. , Seal, M. L. , Josev, E. K. , Nosarti, C. , Gathercole, S. , & Anderson, P. J. (2013). Preventing academic difficulties in preterm children: A randomised controlled trial of an adaptive working memory training intervention—IMPRINT study. BMC Pediatrics, 13(1), 144. 10.1186/1471-2431-13-144 24041245 PMC3848656

[cdev14180-bib-0042] Pelegrina, S. , Lechuga, M. T. , García‐Madruga, J. A. , Elosúa, M. R. , Macizo, P. , Carreiras, M. , Fuentes, L. J. , & Bajo, M. T. (2015). Normative data on the n‐back task for children and young adolescents. Frontiers in Psychology, 6, 1544. 10.3389/fpsyg.2015.01544 26500594 PMC4597481

[cdev14180-bib-0043] Peterson, W. W. , Birdsall, T. G. , & Fox, W. C. (1954). The theory of signal dectectability. IRE Professional Group on Information Theory, 4(4), 171–212. 10.1109/TIT.1954.1057460

[cdev14180-bib-0044] R Core Team . (2022). R: A language and environment for statistical computing (4.2.0). R Foundation for Statistical Computing.

[cdev14180-bib-0045] Raven, J. , Raven, J. C. , & Court, J. H. (2000). Manual for raven's progressive matrices and vocabulary scales. Section 3: The Standard Progressive Matrices. Psychology Press

[cdev14180-bib-0046] Redick, T. S. (2019). The hype cycle of working memory training. Current Directions in Psychological Science, 28(5), 423–429. 10.1177/0963721419848668 31814661 PMC6897530

[cdev14180-bib-0047] Roberts, G. , Howard, K. , Spittle, A. J. , Brown, N. C. , Anderson, P. J. , & Doyle, L. W. (2008). Rates of early intervention services in very preterm children with developmental disabilities at age 2 years. Journal of Paediatrics and Child Health, 44(5), 276–280. 10.1111/j.1440-1754.2007.01251.x 17999667

[cdev14180-bib-0048] Roberts, G. , Quach, J. , Spencer‐Smith, M. , Anderson, P. J. , Gathercole, S. , Gold, L. , Sia, K. L. , Mensah, F. , Rickards, F. , Ainley, J. , & Wake, M. (2016). Academic outcomes 2 years after working memory training for children with lowworking memory: A randomized clinical trial. JAMA Pediatrics, 170(5), e154568. 10.1001/jamapediatrics.2015.4568 26954779

[cdev14180-bib-0049] RStudio Team . (2022). RStudio: Integrated Development for R. RStudio, PBC.

[cdev14180-bib-0050] Ryan, R. M. , & Deci, E. L. (2000a). Intrinsic and extrinsic motivations: Classic definitions and new directions. Contemporary Educational Psychology, 25(1), 54–67. 10.1006/ceps.1999.1020 10620381

[cdev14180-bib-0051] Ryan, R. M. , & Deci, E. L. (2000b). Self‐determination theory and the facilitation of intrinsic motivation, social development, and well‐being. American Psychologist, 55(1), 68–78. 10.1037/0003-066X.55.1.68 11392867

[cdev14180-bib-0052] Sala, G. , & Gobet, F. (2017). Working memory training in typically developing children: A meta‐analysis of the available evidence. Developmental Psychology, 53(4), 671–685. 10.1037/dev0000265 28165253

[cdev14180-bib-0053] Sala, G. , & Gobet, F. (2020). Working memory training in typically developing children: A multilevel meta‐analysis. Psychonomic Bulletin and Review, 27(3), 423–434. 10.3758/s13423-019-01681-y 31939109

[cdev14180-bib-0054] Schulz, K. F. , Altman, D. G. , & Moher, D. (2010). CONSORT 2010 statement: Updated guidelines for reporting parallel group randomised trials. BMC Medicine, 8(1), 1–9. 10.1186/1741-7015-8-18/FIGURES/1 20334633 PMC2860339

[cdev14180-bib-0055] Schulz, K. F. , & Grimes, D. A. (2002). Sample size slippages in randomised trials: Exclusions and the lost and wayward [reprint in *Z Arztl Fortbild Qualitatssich*. 2006;100(6):467–73; PMID: 17058892]. Lancet, 359(9308), 781–785. 11888606 10.1016/S0140-6736(02)07882-0

[cdev14180-bib-0056] Simons, D. J. , Boot, W. R. , Charness, N. , Gathercole, S. E. , Chabris, C. F. , Hambrick, D. Z. , & Stine‐Morrow, E. A. L. (2016). Do “brain‐training” programs work? Psychological Science in the Public Interest, 17(3), 103–186. 10.1177/1529100616661983 27697851

[cdev14180-bib-0057] Soveri, A. , Antfolk, J. , Karlsson, L. , Salo, B. , & Laine, M. (2017). Working memory training revisited: A multi‐level meta‐analysis of n‐back training studies. Psychonomic Bulletin and Review, 24(4), 1077–1096. 10.3758/s13423-016-1217-0 28116702

[cdev14180-bib-0058] Steyvers, M. , & Schafer, R. J. (2020). Inferring latent learning factors in large‐scale cognitive training data. Nature Human Behaviour, 4(11), 1145–1155. 10.1038/s41562-020-00935-3 32868884

[cdev14180-bib-0059] Strauss, E. , Sherman, E. M. , & Spreen, O. (2006). A compendium of neuropsychological tests: Administration, norms, and commentary (3rd ed.). Oxford University Press. 10.1212/wnl.41.11.1856-a

[cdev14180-bib-0060] Szmalec, A. , Verbruggen, F. , Vandierendonck, A. , & Kemps, E. (2011). Control of interference during working memory updating. Journal of Experimental Psychology: Human Perception and Performance, 37(1), 137–151. 10.1037/a0020365 20731517

[cdev14180-bib-0061] Thorell, L. B. , Lindqvist, S. , Nutley, S. B. , Bohlin, G. , & Klingberg, T. (2009). Training and transfer effects of executive functions in preschool children. Developmental Science, 12(1), 106–113. 10.1111/j.1467-7687.2008.00745.x 19120418

[cdev14180-bib-0062] Tidwell, J. W. , Dougherty, M. R. , Chrabaszcz, J. R. , Thomas, R. P. , & Mendoza, J. L. (2014). What counts as evidence for working memory training? Problems with correlated gains and dichotomization. Psychonomic Bulletin and Review, 21(3), 620–628. 10.3758/s13423-013-0560-7 24307249

[cdev14180-bib-0063] von Bastian, C. C. , Belleville, S. , Udale, R. C. , Reinhartz, A. , Essounni, M. , & Strobach, T. (2022). Mechanisms underlying training‐induced cognitive change. Nature Reviews Psychology, 1(1), 30–41. 10.1038/s44159-021-00001-3 PMC9270821 35804410

[cdev14180-bib-0064] von Bastian, C. C. , & Eschen, A. (2016). Does working memory training have to be adaptive? Psychological Research, 80(2), 181–194. 10.1007/s00426-015-0655-z 25716189

[cdev14180-bib-0065] von Bastian, C. C. , & Oberauer, K. (2014). Effects and mechanisms of working memory training: A review. Psychological Research, 78(6), 803–820. 10.1007/s00426-013-0524-6 24213250

[cdev14180-bib-0066] Vygotsky, L. S. (1978). Interaction between learning and development. In M. Cole , V. Jolm‐Steiner , S. Scribne , & E. Souberman (Eds.), Mind in society: The development of higher psychological processes development of higher psychological processes (pp. 79–91). Harvard University Press.

[cdev14180-bib-0067] Wechsler, D. (2014). WISC‐V: Technical and interpretive manual. Pearson.

[cdev14180-bib-0068] Westerberg, H. , & Klingberg, T. (2007). Changes in cortical activity after training of working memory—A single‐subject analysis. Physiology and Behavior, 92(1–2), 186–192. 10.1016/J.PHYSBEH.2007.05.041 17597168

