# Supplementary material for: Does working memory training in children need to be adaptive? A randomized controlled trial
Source: Child Dev. 2024 Oct 23;96(2):508–26. doi: 10.1111/cdev.14180 (PMC11868676; doi:10.1111/cdev.14180)
Supplement: Supplementary file 1 — Data S1. [file CDEV-96-508-s001.docx]

**Supporting Information**

**Table S1.** The number of children for each means and standard deviation presented in Table 3.

|  | Baseline | | | |  | Immediately post-intervention | | | |  | 6 month post-intervention | | | |
| --- | --- | --- | --- | --- | --- | --- | --- | --- | --- | --- | --- | --- | --- | --- |
|  | Active Control  (n = 50)^a^ | Adaptive Training  (n = 63)^a^ | Self-select  Training  (n = 37)^a^ | Stepwise  Training  (n = 51)^a^ |  | Active Control  (n = 48)^a^ | Adaptive  Training  (n = 57)^a^ | Self-select  Training  (n = 36)^a^ | Stepwise  Training  (n = 50)^a^ |  | Active Control  (n = 49)^a^ | Adaptive  Training  (n = 60)^a^ | Self-select  Training  (n = 36)^a^ | Stepwise  Training  (n = 50)^a^ |
| **Near Transfer** | | | | | | | | | | | | | | |
| BS digits | 50 | 63 | 37 | 51 |  | 48 | 57 | 36 | 50 |  | 49 | 60 | 36 | 50 |
| FI objects | 50 | 63 | 36^b^ | 51 |  | 48 | 57 | 36 | 50 |  | 49 | 60 | 36 | 50 |
| BS letters | 50 | 63 | 37 | 51 |  | 48 | 57 | 36 | 50 |  | 49 | 60 | 36 | 50 |
| FI letters | 49^b^ | 62^b^ | 37 | 51 |  | 48 | 56^b^ | 36 | 50 |  | 49 | 60 | 36 | 50 |
| **Intermediate transfer** | | | | | | | | | | | | | | |
| 2-back objects^c^ | 50 | 63 | 37 | 51 |  | 47 | 57 | 36 | 50 |  | 49 | 60 | 36 | 50 |
| **Far transfer** | | | | | | | | | | | | | | |
| Reasoning^d^ | 50 | 63 | 37 | 51 |  | 48 | 57 | 36 | 50 |  | 49 | 60 | 36 | 50 |
| Inattention^e^ | 34 | 43 | 31 | 36 |  | 33 | 32 | 22 | 32 |  | 22 | 27 | 15 | 26 |
| Hyperactivity^e^ | 34 | 43 | 31 | 36 |  | 33 | 32 | 22 | 32 |  | 22 | 27 | 15 | 26 |

BS = Backward span. FI = Following instructions.

^a^ Number of children assessed per time point and condition (corresponds to Figure 1, CONSORT diagram).

^b^ Child did not want to complete this test.

^c^ Number of children for the number of hits, correct rejections, false alarms, no responses, and d-prime (*d’*).

^d^ Raven’s SPM sets A and B score.

^e^ ADHD-RS-5 score.

**Table S2.** Additional statistics (omnibus tests and variance explained) for the multiple regression analyses presented in Table 4.

| Transfer Range / Parameter | Immediately Post-intervention | | | |  | 6 months Post-intervention | | | |
| --- | --- | --- | --- | --- | --- | --- | --- | --- | --- |
|  | *F* | *df* | *p* | R^2^ |  | *F* | *df* | *p* | R^2^ |
| **Near transfer - backward span digits** |  |  |  |  |  |  |  |  |  |
| Overall model | 14.72 | 6, 184 | >.001 | 0.32 |  | 8.20 | 6,188 | >.001 | 0.21 |
| **Near transfer - following instructions objects** |  |  |  |  |  |  |  |  |  |
| Overall model | 10.34 | 6, 184 | >.001 | 0.25 |  | 7.67 | 6,187 | >.001 | 0.20 |
| **Near transfer - backward span letters** |  |  |  |  |  |  |  |  |  |
| Overall model | 14.58 | 6,184 | >.001 | 0.32 |  | 6.55 | 6,188 | >.001 | 0.17 |
| **Near transfer - following instructions letters** |  |  |  |  |  |  |  |  |  |
| Overall model | 5.83 | 6,181 | >.001 | 0.16 |  | 4.56 | 6,186 | >.001 | 0.13 |
| **Intermediate transfer - 2-back objects** |  |  |  |  |  |  |  |  |  |
| Overall model | 5.66 | 6,183 | >.001 | 0.16 |  | 3.54 | 6,188 | .002 | 0.10 |
| **Far transfer – reasoning^a^** |  |  |  |  |  |  |  |  |  |
| Overall model | 45.29 | 6, 184 | >.001 | 0.60 |  | 20.98 | 6,188 | >.001 | 0.40 |
| **Far transfer – inattention^b^** |  |  |  |  |  |  |  |  |  |
| Overall model | 63.16 | 6,104 | >.001 | 0.78 |  | 45.64 | 6,79 | >.001 | 0.78 |
| **Far transfer – hyperactivity^b^** |  |  |  |  |  |  |  |  |  |
| Overall model | 36.69 | 6,104 | >.001 | 0.68 |  | 31.56 | 6.79 | >.001 | 0.71 |

^a^ Raven’s SPM sets A and B score. ^b^ ADHD-RS-5 score.

**Table S3.** Exploratory ancillary analyses: comparisons from baseline to immediately post-intervention, and baseline to 6-months post-intervention while controlling for group-differences in child motivation (relevant rows are in italics).

| Transfer Range / Parameter | Immediately Post-intervention | | |  | 6 months Post-intervention | | |
| --- | --- | --- | --- | --- | --- | --- | --- |
|  | Est. [95% CI] | *p* | *f* ^2^ |  | Est. [95% CI] | *p* | *f* ^2^ |
| **Near transfer - backward span digits** |  |  |  |  |  |  |  |
| Intercept | 1.84[-2.59,6.28] | .413 |  |  | 4.17[-0.85,9.19] | .103 |  |
| Baseline scores | 0.6[0.45,0.76] | <.001 | 0.32 |  | 0.44[0.26,0.61] | <.001 | 0.14 |
| Stratified age groups |  |  | 0.07 |  |  |  | 0.04 |
| 9-10 years vs. 7-8 years | 1.38[0.42,2.35] | .005 |  |  | 1.38[0.28,2.48] | .014 |  |
| 11-12 years vs. 7-8 years | 2.97[0.84,5.11] | .007 |  |  | 2.28[-0.12,4.69] | .063 |  |
| Condition | -0.09[-1.36,1.18] | .889 | 0.00 |  |  |  | 0.01 |
| Adaptive vs. active control | 0.35[-1.07,1.76] | .629 |  |  | -0.42[-1.86,1.01] | .560 |  |
| Self-select vs. active control | 0.23[-1.07,1.53] | .723 |  |  | -0.65[-2.26,0.96] | .427 |  |
| Stepwise vs. active control |  |  |  |  | -0.81[-2.28,0.65] | .276 |  |
| *Baseline curiosity^a^* | *-0.14[-0.36,0.09]* | *.238* | *0.01* |  | *0.16[-0.1,0.41]* | *.229* | *0.01* |
| *Training interest/enjoyment^b^* | *0.11[-0.07,0.29]* | *.225* | *0.01* |  | *-0.1[-0.3,0.11]* | *.359* | *0.00* |
| Overall model | *F*(8, 180) = 11.06, *p* <.001, R^2^ = 0.33 | | |  | *F*(8, 176) = 6.11, *p* <.001, R^2^ = 0.22 | | |
| **Near transfer - following instructions objects** | |  |  |  |  |  |  |
| Intercept | 2.36[-0.66,5.38] | .124 |  |  | 1.46[-2.04,4.95] | .412 |  |
| Baseline scores | 0.32[0.18,0.46] | .000 | 0.11 |  | 0.38[0.21,0.55] | <.001 | 0.12 |
| Stratified age groups |  |  | 0.10 |  |  |  | 0.04 |
| 9-10 years vs. 7-8 years | 1.3[0.65,1.94] | <.001 |  |  | 0.88[0.13,1.64] | .022 |  |
| 11-12 years vs. 7-8 years | 2.01[0.58,3.45] | .006 |  |  | 1.59[-0.07,3.24] | .060 |  |
| Condition |  |  | 0.02 |  |  |  | 0.01 |
| Adaptive vs. active control | 0.34[-0.49,1.17] | .418 |  |  | -0.07[-1.03,0.88] | .879 |  |
| Self-select vs. active control | 0.84[-0.09,1.77] | .077 |  |  | 0.49[-0.6,1.57] | .377 |  |
| Stepwise vs. active control | 0.49[-0.36,1.34] | .259 |  |  | -0.01[-1,0.97] | .980 |  |
| *Baseline curiosity^a^* | *-0.01[-0.16,0.14]* | *.922* | *0.00* |  | *0.13[-0.04,0.3]* | *.130* | *0.01* |
| *Training interest/enjoyment^b^* | *0.06[-0.06,0.18]* | *.306* | *0.01* |  | *0.05[-0.09,0.18]* | *.511* | *0.00* |
| Overall model | *F*(8, 179) = 7.76, *p* <.001, R^2^ = 0.26 | | |  | *F*(8, 175) = 5.74, *p* <.001, R^2^ = 0.21 | | |

^a^ Intrinsic Motivation Scale (IMS). ^b^ Intrinsic Motivation Inventory (IMI).

**Table S3.** (continued).

| Transfer Range / Parameter | Immediately Post-intervention | | |  | 6 months Post-intervention | | |
| --- | --- | --- | --- | --- | --- | --- | --- |
|  | Est. [95% CI] | *p* | *f* ^2^ |  | Est. [95% CI] | *p* | *f* ^2^ |
| **Near transfer - backward span letters** |  |  |  |  |  |  |  |
| Intercept | 2.16[-1.21,5.52] | .207 |  |  | 4.46[0.9,8.02] | .014 |  |
| Baseline scores | 0.49[0.34,0.64] | .000 | 0.23 |  | 0.32[0.16,0.48] | <.001 | 0.09 |
| Stratified age groups |  |  | 0.09 |  |  |  | 0.05 |
| 9-10 years vs. 7-8 years | 1.03[0.28,1.77] | .007 |  |  | 0.61[-0.18,1.4] | .131 |  |
| 11-12 years vs. 7-8 years | 2.98[1.37,4.6] | <.001 |  |  | 2.61[0.91,4.31] | .003 |  |
| Condition |  |  | 0.00 |  |  |  | 0.01 |
| Adaptive vs. active control | -0.2[-1.15,0.76] | .683 |  |  | -0.04[-1.05,0.96] | .930 |  |
| Self-select vs. active control | -0.37[-1.45,0.7] | .493 |  |  | 0.46[-0.68,1.6] | .428 |  |
| Stepwise vs. active control | -0.03[-1.01,0.96] | .957 |  |  | -0.34[-1.38,0.7] | .521 |  |
| *Baseline curiosity^a^* | *0.11[-0.06,0.28]* | *.206* | *0.01* |  | *0.16[-0.02,0.34]* | *.090* | *0.02* |
| *Training interest/enjoyment^b^* | *-0.05[-0.18,0.09]* | *.500* | *0.00* |  | *-0.14[-0.28,0.01]* | *.067* | *0.02* |
| Overall model | *F*(8, 180) = 10.6, *p* <.001, R^2^ = 0.32 | | |  | *F*(8, 176) = 5.36, *p* <.001, R^2^ = 0.20 | | |
| **Near transfer - following instructions letters** | |  |  |  |  |  |  |
| Intercept | 2.2[-0.71,5.12] | .137 |  |  | 5.3[2.62,7.99] | <.001 |  |
| Baseline scores | 0.35[0.18,0.52] | .000 | 0.10 |  | 0.27[0.11,0.42] | .001 | 0.07 |
| Stratified age groups |  |  | 0.04 |  |  |  | 0.06 |
| 9-10 years vs. 7-8 years | 0.77[0.17,1.36] | .012 |  |  | 0.82[0.27,1.37] | .004 |  |
| 11-12 years vs. 7-8 years | 1.23[-0.11,2.56] | .072 |  |  | -0.38[-1.6,0.85] | .545 |  |
| Condition |  |  | 0.01 |  |  |  | 0.02 |
| Adaptive vs. active control | -0.26[-1.06,0.54] | .526 |  |  | 0.15[-0.58,0.89] | .683 |  |
| Self-select vs. active control | -0.34[-1.23,0.55] | .452 |  |  | 0.24[-0.59,1.06] | .573 |  |
| Stepwise vs. active control | -0.5[-1.32,0.31] | .224 |  |  | -0.43[-1.18,0.32] | .258 |  |
| *Baseline curiosity^a^* | *0.09[-0.05,0.23]* | *.195* | *0.01* |  | *-0.05[-0.18,0.08]* | *.413* | *0.00* |
| *Training interest/enjoyment^b^* | *0[-0.12,0.11]* | *.988* | *0.00* |  | *-0.03[-0.13,0.08]* | *.633* | *0.00* |
| Overall model | *F*(8, 177) = 4.14, *p* <.001, R^2^ = 0.16 | | |  | *F*(8, 174) = 4.00, *p* <.001, R^2^ = 0.16 | | |

^a^ Intrinsic Motivation Scale (IMS). ^b^ Intrinsic Motivation Inventory (IMI).

**Table S3.** (continued).

| Transfer Range / Parameter | Immediately Post-intervention | | |  | 6 months Post-intervention | | |
| --- | --- | --- | --- | --- | --- | --- | --- |
|  | Est. [95% CI] | *p* | *f* ^2^ |  | Est. [95% CI] | *p* | *f* ^2^ |
| **Intermediate transfer - 2-back objects** |  |  |  |  |  |  |  |
| Intercept | 1.79[0.64,2.93] | .002 |  |  | 1.46[0.41,2.51] | .007 |  |
| Baseline scores | 0.19[0.05,0.33] | .009 | 0.04 |  | 0.18[0.05,0.31] | .006 | 0.04 |
| Stratified age groups |  |  | 0.09 |  |  |  | 0.03 |
| 9-10 years vs. 7-8 years | 0.5[0.25,0.75] | <.001 |  |  | 0.29[0.05,0.52] | .016 |  |
| 11-12 years vs. 7-8 years | 0.55[-0.02,1.13] | .059 |  |  | 0.28[-0.25,0.8] | .298 |  |
| Condition |  |  | 0.01 |  |  |  | 0.01 |
| Adaptive vs. active control | 0.01[-0.33,0.34] | .976 |  |  | -0.11[-0.41,0.19] | .477 |  |
| Self-select vs. active control | -0.1[-0.47,0.28] | .610 |  |  | -0.13[-0.47,0.21] | .448 |  |
| Stepwise vs. active control | 0.17[-0.17,0.51] | .330 |  |  | -0.19[-0.5,0.12] | .228 |  |
| *Baseline curiosity^a^* | *-0.05[-0.11,0.01]* | *.104* | *0.01* |  | *0.03[-0.03,0.08]* | *.353* | *0.00* |
| *Training interest/enjoyment^b^* | *0[-0.04,0.05]* | *.924* | *0.00* |  | *-0.02[-0.06,0.03]* | *.481* | *0.00* |
| Overall model | *F*(8, 179) = 4.53, *p* <.001, R^2^ = 0.17 | | |  | *F*(8, 176) = 2.67, *p* = .009, R^2^ = 0.11 | | |
| **Far transfer – reasoning^c^** |  |  |  |  |  |  |  |
| Intercept | 5.06[1.75,8.36] | .003 |  |  | 8.96[5.46,12.45] | <.001 |  |
| Baseline scores | 0.73[0.63,0.83] | .000 | 1.11 |  | 0.51[0.4,0.62] | <.001 | 0.47 |
| Stratified age groups |  |  | 0.00 |  |  |  | 0.01 |
| 9-10 years vs. 7-8 years | 0.01[-0.68,0.7] | .973 |  |  | -0.41[-1.14,0.33] | .280 |  |
| 11-12 years vs. 7-8 years | 0.27[-1.2,1.74] | .716 |  |  | 0.49[-1.06,2.03] | .534 |  |
| Condition |  |  | 0.01 |  |  |  | 0.02 |
| Adaptive vs. active control | 0.54[-0.31,1.39] | .212 |  |  | 0.25[-0.64,1.15] | .577 |  |
| Self-select vs. active control | 0.43[-0.52,1.38] | .376 |  |  | 0.82[-0.19,1.83] | .110 |  |
| Stepwise vs. active control | 0.3[-0.58,1.18] | .500 |  |  | -0.17[-1.09,0.75] | .721 |  |
| *Baseline curiosity^a^* | *0.05[-0.1,0.2]* | *.538* | *0.00* |  | *0.05[-0.11,0.21]* | *.527* | *0.00* |
| *Training interest/enjoyment^b^* | *-0.02[-0.14,0.1]* | *.761* | *0.00* |  | *0.02[-0.11,0.15]* | *.753* | *0.00* |
| Overall model | *F*(8, 180) = 31.99, *p* <.001, R^2^ = 0.59 | | |  | *F*(8, 176) = 13.1, *p* < .001, R^2^ = 0.37 | | |

^a^ Intrinsic Motivation Scale (IMS). ^b^ Intrinsic Motivation Inventory (IMI). ^c^ Raven’s SPM sets A and B score.

**Table S3.** (continued).

| Transfer Range / Parameter | Immediately Post-intervention | | |  | 6 months Post-intervention | | |
| --- | --- | --- | --- | --- | --- | --- | --- |
|  | Est. [95% CI] | *p* | *f* ^2^ |  | Est. [95% CI] | *p* | *f* ^2^ |
| **Far transfer – inattention^d^** |  |  |  |  |  |  |  |
| Intercept | -1.23[-5.23,2.77] | .544 |  |  | 0.52[-4.77,5.82] | .845 |  |
| Baseline scores | 0.76[0.67,0.84] | <.001 | 3.37 |  | 0.73[0.64,0.83] | <.001 | 3.29 |
| Stratified age groups | 0[0,0] |  | 0.04 |  |  |  | 0.03 |
| 9-10 years vs. 7-8 years | -0.96[-1.92,0.01] | .052 |  |  | -0.74[-1.91,0.43] | .213 |  |
| 11-12 years vs. 7-8 years | -1.16[-3.02,0.7] | .220 |  |  | -1.39[-3.67,0.88] | .225 |  |
| Condition |  |  | 0.03 |  |  |  | 0.01 |
| Adaptive vs. active control | -0.56[-1.86,0.74] | .392 |  |  | -0.09[-1.7,1.51] | .907 |  |
| Self-select vs. active control | -0.97[-2.39,0.45] | .177 |  |  | -0.66[-2.39,1.07] | .447 |  |
| Stepwise vs. active control | -0.99[-2.28,0.29] | .129 |  |  | 0[-1.64,1.63] | .999 |  |
| *Baseline curiosity^a^* | *0.2[-0.04,0.43]* | *.100* | *0.03* |  | *-0.01[-0.28,0.26]* | *.941* | *0.00* |
| *Training interest/enjoyment^b^* | *0.05[-0.12,0.22]* | *.591* | *0.00* |  | *0.06[-0.2,0.32]* | *.647* | *0.00* |
| Overall model | *F*(8, 98) = 47.43, *p* <.001, R^2^ = 0.79 | | |  | *F*(8, 71) = 33.75, *p* < .001, R^2^ = 0.79 | | |
| **Far transfer – hyperactivity^d^** |  |  |  |  |  |  |  |
| Intercept | -0.63[-4.37,3.1] | .737 |  |  | 0.87[-3.83,5.57] | .714 |  |
| Baseline scores | 0.75[0.63,0.86] | .000 | 1.77 |  | 0.8[0.67,0.92] | <.001 | 2.36 |
| Stratified age groups |  |  | 0.01 |  |  |  | 0.00 |
| 9-10 years vs. 7-8 years | -0.29[-1.21,0.63] | .530 |  |  | 0.3[-0.76,1.36] | .577 |  |
| 11-12 years vs. 7-8 years | -0.62[-2.4,1.16] | .490 |  |  | -0.01[-2.06,2.04] | .993 |  |
| Condition |  |  | 0.04 |  |  |  | 0.03 |
| Adaptive vs. active control | -0.73[-1.95,0.5] | .241 |  |  | -0.48[-1.91,0.95] | .507 |  |
| Self-select vs. active control | 0.46[-0.88,1.81] | .494 |  |  | -1.15[-2.69,0.38] | .138 |  |
| Stepwise vs. active control | 0.01[-1.21,1.23] | .989 |  |  | -0.5[-1.95,0.96] | .499 |  |
| *Baseline curiosity^a^* | *-0.04[-0.26,0.18]* | *.725* | *0.00* |  | *0.04[-0.21,0.28]* | *.755* | *0.00* |
| *Training interest/enjoyment^b^* | *0.11[-0.05,0.27]* | *.190* | *0.02* |  | *-0.04[-0.27,0.2]* | *.745* | *0.00* |
| Overall model | *F*(8, 98) = 26.59, *p* <.001, R^2^ = 0.68 | | |  | *F*(8, 71) = 24.01, *p* < .001, R^2^ = 0.73 | | |

^a^ Intrinsic Motivation Scale (IMS). ^b^ Intrinsic Motivation Inventory (IMI). ^d^ ADHD-RS-5 score.

**Table S4.** Exploratory ancillary analyses: comparisons from baseline to immediately post-intervention, and baseline to 6-months post-intervention while exploring differential effects of child sex (relevant rows are in italics).

| Transfer Range / Parameter | Immediately Post-intervention | | |  | 6 months Post-intervention | | |
| --- | --- | --- | --- | --- | --- | --- | --- |
|  | Est. [95% CI] | *p* | *f* ^2^ |  | Est. [95% CI] | *p* | *f* ^2^ |
| **Near transfer - backward span digits** |  |  |  |  |  |  |  |
| Intercept | 2.08[0.62,3.54] | .005 |  |  | 4.77[3.17,6.36] | <.001 |  |
| Baseline scores | 0.58[0.42,0.73] | <.001 | 0.29 |  | 0.44[0.27,0.61] | <.001 | 0.14 |
| Stratified age groups |  |  | 0.06 |  |  |  | 0.05 |
| 9-10 years vs. 7-8 years | 1.38[0.42,2.34] | .005 |  |  | 1.4[0.34,2.47] | .010 |  |
| 11-12 years vs. 7-8 years | 2.71[0.56,4.86] | .014 |  |  | 2.28[0.07,4.49] | .043 |  |
| Condition |  |  | 0.01 |  |  |  | 0.01 |
| Adaptive vs. active control | -0.73[-2.37,0.9] | .377 |  |  | -0.63[-2.41,1.14] | .483 |  |
| Self-select vs. active control | 0.68[-1.34,2.7] | .506 |  |  | -1.05[-3.29,1.19] | .355 |  |
| Stepwise vs. active control | 0.28[-1.5,2.05] | .760 |  |  | -1.39[-3.33,0.55] | .158 |  |
| *Sex^a^* |  |  | *0.00* |  |  |  | *0.00* |
| *Female vs. male* | *0.16[-1.73,2.04]* | *.871* |  |  | *-0.76[-2.85,1.33]* | *.473* |  |
| *Condition*Sex* |  |  | *0.01* |  |  |  | *0.01* |
| *Adaptive*Female* | *1.14[-1.37,3.64]* | *.372* |  |  | *1[-1.74,3.74]* | *.473* |  |
| *Self-select*Female* | *-0.84[-3.68,2]* | *.561* |  |  | *1.19[-1.96,4.34]* | *.457* |  |
| *Stepwise*Female* | *-0.35[-2.94,2.25]* | *.793* |  |  | *1.84[-1.04,4.72]* | *.208* |  |
| Overall model | *F*(10, 180) = 9.06, *p* <.001, R^2^ = 0.33 | | |  | *F*(10, 180) = 9.06, *p* <.001, R^2^ = 0.33 | | |

^a^ Reference group = Male.

**Table S4.** (continued).

| Transfer Range / Parameter | Immediately Post-intervention | | |  | 6 months Post-intervention | | |
| --- | --- | --- | --- | --- | --- | --- | --- |
|  | Est. [95% CI] | *p* | *f* ^2^ |  | Est. [95% CI] | *p* | *f* ^2^ |
| **Near transfer - following instructions objects** |  |  |  |  |  |  |  |
| Intercept | 3.5[2.42,4.57] | <.001 |  |  | 4.13[2.87,5.39] | <.001 |  |
| Baseline scores | 0.31[0.17,0.45] | <.001 | 0.10 |  | 0.34[0.17,0.51] | <.001 | 0.09 |
| Stratified age groups |  |  | 0.10 |  |  |  | 0.06 |
| 9-10 years vs. 7-8 years | 1.29[0.65,1.94] | <.001 |  |  | 1.06[0.32,1.8] | .005 |  |
| 11-12 years vs. 7-8 years | 2.06[0.62,3.51] | .005 |  |  | 2[0.46,3.54] | .011 |  |
| Condition |  |  | 0.02 |  |  |  | 0.01 |
| Adaptive vs. active control | 0.67[-0.4,1.73] | .216 |  |  | 0.57[-0.64,1.77] | .355 |  |
| Self-select vs. active control | 1.13[-0.22,2.47] | .101 |  |  | 0.48[-1.08,2.04] | .546 |  |
| Stepwise vs. active control | 0.53[-0.62,1.68] | .366 |  |  | 0.27[-1.04,1.59] | .684 |  |
| *Sex^a^* |  |  | *0.00* |  |  |  | *0.01* |
| *Female vs. male* | *0.04[-1.17,1.24]* | *.954* |  |  | *0.78[-0.62,2.18]* | *.273* |  |
| *Condition*Sex* |  |  | *0.01* |  |  |  | *0.01* |
| *Adaptive*Female* | *-0.76[-2.39,0.86]* | *.356* |  |  | *-1.39[-3.25,0.46]* | *.141* |  |
| *Self-select*Female* | *-0.63[-2.49,1.23]* | *.504* |  |  | *-0.29[-2.45,1.87]* | *.791* |  |
| *Stepwise*Female* | *-0.09[-1.76,1.58]* | *.917* |  |  | *-0.76[-2.7,1.18]* | *.440* |  |
| Overall model | *F*(10, 179) = 6.40, *p* <.001, R^2^ = 0.26 | | |  | *F*(10, 183) = 4.82, *p* <.001, R^2^ = 0.21 | | |

^a^ Reference group = Male.

**Table S4.** (continued).

| Transfer Range / Parameter | Immediately Post-intervention | | |  | 6 months Post-intervention | | |
| --- | --- | --- | --- | --- | --- | --- | --- |
|  | Est. [95% CI] | *p* | *f* ^2^ |  | Est. [95% CI] | *p* | *f* ^2^ |
| **Near transfer - backward span letters** |  |  |  |  |  |  |  |
| Intercept | 2.49[1.35,3.62] | <.001 |  |  | 3.81[2.64,4.98] | <.001 |  |
| Baseline scores | 0.5[0.35,0.65] | <.001 | 0.24 |  | 0.32[0.16,0.48] | <.001 | 0.09 |
| Stratified age groups |  |  | 0.10 |  |  |  | 0.06 |
| 9-10 years vs. 7-8 years | 1.07[0.32,1.82] | .005 |  |  | 0.57[-0.2,1.33] | .149 |  |
| 11-12 years vs. 7-8 years | 3.16[1.53,4.8] | <.001 |  |  | 2.61[1.05,4.17] | .001 |  |
| Condition |  |  | 0.00 |  |  |  | 0.00 |
| Adaptive vs. active control | 0.06[-1.17,1.3] | .918 |  |  | -0.07[-1.31,1.17] | .913 |  |
| Self-select vs. active control | -0.59[-2.12,0.94] | .449 |  |  | 0.41[-1.17,1.99] | .611 |  |
| Stepwise vs. active control | -0.02[-1.36,1.33] | .979 |  |  | -0.09[-1.45,1.28] | .899 |  |
| *Sex^a^* |  |  | *0.00* |  |  |  | *0.00* |
| *Female vs. male* | *0.64[-0.77,2.05]* | *.370* |  |  | *0.68[-0.77,2.14]* | *.355* |  |
| *Condition*Sex* |  |  | *0.00* |  |  |  | *0.00* |
| *Adaptive*Female* | *-0.39[-2.29,1.51]* | *.686* |  |  | *0.27[-1.66,2.2]* | *.780* |  |
| *Self-select*Female* | *0.33[-1.81,2.48]* | *.761* |  |  | *0.33[-1.88,2.55]* | *.767* |  |
| *Stepwise*Female* | *-0.07[-2.02,1.88]* | *.943* |  |  | *-0.19[-2.21,1.82]* | *.850* |  |
| Overall model | *F*(10, 180) = 9.01, *p* <.001, R^2^ = 0.33 | | |  | *F*(10, 184) = 4.45, *p* <.001, R^2^ = 0.19 | | |

^a^ Reference group = Male.

**Table S4.** (continued).

| Transfer Range / Parameter | Immediately Post-intervention | | |  | 6 months Post-intervention | | |
| --- | --- | --- | --- | --- | --- | --- | --- |
|  | Est. [95% CI] | *p* | *f* ^2^ |  | Est. [95% CI] | *p* | *f* ^2^ |
| **Near transfer - following instructions letters** |  |  |  |  |  |  |  |
| Intercept | 3.49[2.28,4.7] | <.001 |  |  | 4.03[2.84,5.21] | <.001 |  |
| Baseline scores | 0.36[0.2,0.53] | <.001 | 0.10 |  | 0.26[0.1,0.42] | .002 | 0.06 |
| Stratified age groups |  |  | 0.04 |  |  |  | 0.04 |
| 9-10 years vs. 7-8 years | 0.76[0.16,1.36] | .013 |  |  | 0.79[0.21,1.37] | .008 |  |
| 11-12 years vs. 7-8 years | 1.2[-0.15,2.55] | .082 |  |  | 0.79[-0.43,2] | .202 |  |
| Condition |  |  | 0.00 |  |  |  | 0.03 |
| Adaptive vs. active control | -0.3[-1.34,0.74] | .574 |  |  | 0.56[-0.43,1.54] | .265 |  |
| Self-select vs. active control | -0.53[-1.8,0.75] | .417 |  |  | 0.31[-0.94,1.56] | .623 |  |
| Stepwise vs. active control | -0.1[-1.22,1.03] | .867 |  |  | -0.64[-1.72,0.44] | .241 |  |
| *Sex^a^* |  |  | *0.00* |  |  |  | *0.00* |
| *Female vs. male* | *-0.27[-1.45,0.9]* | *.646* |  |  | *0.05[-1.1,1.19]* | *.935* |  |
| *Condition*Sex* |  |  | *0.01* |  |  |  | *0.01* |
| *Adaptive*Female* | *0.46[-1.12,2.05]* | *.565* |  |  | *-0.8[-2.32,0.73]* | *.304* |  |
| *Self-select*Female* | *0.55[-1.23,2.33]* | *.542* |  |  | *-0.07[-1.81,1.68]* | *.941* |  |
| *Stepwise*Female* | *-0.3[-1.92,1.32]* | *.714* |  |  | *0.12[-1.46,1.71]* | *.877* |  |
| Overall model | *F*(10, 177) = 3.60, *p* <.001, R^2^ = 0.17 | | |  | *F*(10, 182) = 2.96, *p* = .002, R^2^ = 0.14 | | |

^a^ Reference group = Male.

**Table S4.** (continued).

| Transfer Range / Parameter | Immediately Post-intervention | | |  | 6 months Post-intervention | | |
| --- | --- | --- | --- | --- | --- | --- | --- |
|  | Est. [95% CI] | *p* | *f* ^2^ |  | Est. [95% CI] | *p* | *f* ^2^ |
| **Intermediate transfer - 2-back objects** |  |  |  |  |  |  |  |
| Intercept | 1.13[0.78,1.49] | <.001 |  |  | 1.4[1.09,1.72] | <.001 |  |
| Baseline scores | 0.19[0.05,0.33] | .007 | 0.04 |  | 0.19[0.06,0.31] | .004 | 0.05 |
| Stratified age groups |  |  | 0.10 |  |  |  | 0.03 |
| 9-10 years vs. 7-8 years | 0.53[0.28,0.78] | <.001 |  |  | 0.28[0.05,0.5] | .016 |  |
| 11-12 years vs. 7-8 years | 0.58[0,1.17] | .048 |  |  | 0.21[-0.26,0.69] | .380 |  |
| Condition |  |  | 0.00 |  |  |  | 0.01 |
| Adaptive vs. active control | 0.11[-0.32,0.55] | .602 |  |  | 0[-0.37,0.37] | .994 |  |
| Self-select vs. active control | -0.09[-0.63,0.44] | .728 |  |  | 0.01[-0.47,0.48] | .975 |  |
| Stepwise vs. active control | 0.04[-0.43,0.51] | .857 |  |  | 0.19[-0.22,0.6] | .355 |  |
| *Sex^a^* |  |  | *0.00* |  |  |  | *0.01* |
| *Female vs. male* | *-0.02[-0.51,0.47]* | *.936* |  |  | *0.26[-0.18,0.69]* | *.246* |  |
| *Condition*Sex* |  |  | *0.01* |  |  |  | *0.03* |
| *Adaptive*Female* | *-0.37[-1.03,0.29]* | *.269* |  |  | *-0.17[-0.75,0.41]* | *.562* |  |
| *Self-select*Female* | *-0.05[-0.79,0.7]* | *.904* |  |  | *-0.21[-0.87,0.45]* | *.531* |  |
| *Stepwise*Female* | *0.05[-0.63,0.73]* | *.892* |  |  | *-0.65[-1.25,-0.05]* | *.035^b^* |  |
| Overall model | *F*(10, 179) = 3.70, *p* <.001, R^2^ = 0.17 | | |  | *F*(10, 184) = 2.64, *p* = .005, R^2^ = 0.13 | | |

^a^ Reference group = Male. ^b^ Post-hoc analysis with Tukey’s HSD test to adjust for multiple comparisons not reveal any significant (p <.05) pairwise comparisons for males and females.

**Table S4.** (continued).

| Transfer Range / Parameter | Immediately Post-intervention | | |  | 6 months Post-intervention | | |
| --- | --- | --- | --- | --- | --- | --- | --- |
|  | Est. [95% CI] | *p* | *f* ^2^ |  | Est. [95% CI] | *p* | *f* ^2^ |
| **Far transfer – reasoning^c^** |  |  |  |  |  |  |  |
| Intercept | 5.39[3.54,7.24] | <.001 |  |  | 9.11[7.12,11.1] | <.001 |  |
| Baseline scores | 0.71[0.61,0.81] | <.001 | 1.07 |  | 0.55[0.44,0.66] | <.001 | 0.54 |
| Stratified age groups |  |  | 0.00 |  |  |  | 0.01 |
| 9-10 years vs. 7-8 years | 0.05[-0.63,0.73] | .883 |  |  | -0.49[-1.2,0.22] | .179 |  |
| 11-12 years vs. 7-8 years | 0.34[-1.12,1.8] | .642 |  |  | 0.22[-1.2,1.64] | .765 |  |
| Condition |  |  | 0.02 |  |  |  | 0.03 |
| Adaptive vs. active control | 0.75[-0.33,1.83] | .172 |  |  | 0.4[-0.71,1.51] | .477 |  |
| Self-select vs. active control | -0.07[-1.41,1.27] | .918 |  |  | 1.24[-0.17,2.64] | .084 |  |
| Stepwise vs. active control | 0.78[-0.41,1.97] | .199 |  |  | -0.43[-1.65,0.8] | .491 |  |
| *Sex^a^* |  |  | *0.00* |  |  |  | *0.00* |
| *Female vs. male* | *0.57[-0.67,1.8]* | *.365* |  |  | *0.62[-0.67,1.92]* | *.344* |  |
| *Condition*Sex* |  |  | *0.02* |  |  |  | *0.01* |
| *Adaptive*Female* | *-0.39[-2.06,1.27]* | *.641* |  |  | *-0.25[-1.97,1.47]* | *.773* |  |
| *Self-select*Female* | *0.71[-1.17,2.58]* | *.459* |  |  | *-0.93[-2.9,1.04]* | *.354* |  |
| *Stepwise*Female* | *-0.88[-2.61,0.84]* | *.313* |  |  | *0.18[-1.63,1.99]* | *.847* |  |
| Overall model | *F*(10, 180) = 27.64, *p* <.001, R^2^ = 0.61 | | | | *F*(10, 184) = 12.84, *p* < .001, R^2^ = 0.41 | | |

^a^ Reference group = Male. ^c^ Raven’s SPM sets A and B score.

**Table S4.** (continued).

| Transfer Range / Parameter | Immediately Post-intervention | | |  | 6 months Post-intervention | | |
| --- | --- | --- | --- | --- | --- | --- | --- |
|  | Est. [95% CI] | *p* | *f* ^2^ |  | Est. [95% CI] | *p* | *f* ^2^ |
| **Far transfer – inattention^d^** |  |  |  |  |  |  |  |
| Intercept | 2.74[1.36,4.12] | <.001 |  |  | 1.55[-0.17,3.27] | .077 |  |
| Baseline scores | 0.76[0.67,0.84] | <.001 | 3.15 |  | 0.73[0.63,0.82] | <.001 | 3.20 |
| Stratified age groups |  |  | 0.06 |  |  |  | 0.03 |
| 9-10 years vs. 7-8 years | -1.12[-2.1,-0.14] | .026 |  |  | -0.45[-1.59,0.68] | .429 |  |
| 11-12 years vs. 7-8 years | -1.27[-3.06,0.52] | .162 |  |  | -1.32[-3.28,0.64] | .184 |  |
| Condition |  |  | 0.03 |  |  |  | 0.06 |
| Adaptive vs. active control | -0.95[-2.64,0.74] | .269 |  |  | 0.29[-1.66,2.23] | .770 |  |
| Self-select vs. active control | -1.57[-3.53,0.39] | .115 |  |  | -2.59[-5.41,0.22] | .071 |  |
| Stepwise vs. active control | -1.17[-2.85,0.52] | .173 |  |  | -0.17[-2.15,1.81] | .866 |  |
| *Sex^a^* |  |  | *0.00* |  |  |  | *0.00* |
| *Female vs. male* | *-0.53[-2.33,1.26]* | *.558* |  |  | *-0.36[-2.59,1.88]* | *.751* |  |
| *Condition*Sex* |  |  | *0.01* |  |  |  | *0.07* |
| *Adaptive*Female* | *1.08[-1.49,3.65]* | *.407* |  |  | *-0.97[-3.94,1.99]* | *.515* |  |
| *Self-select*Female* | *0.95[-1.83,3.72]* | *.499* |  |  | *2.86[-0.75,6.47]* | *.119* |  |
| *Stepwise*Female* | *0.63[-1.88,3.13]* | *.621* |  |  | *0.38[-2.6,3.37]* | *.798* |  |
| Overall model | *F*(10, 100) = 36.83, *p* <.001, R^2^ = 0.79 | | | | *F*(10, 75) = 28.29, *p* < .001, R^2^ = 0.79 | | |

^a^ Reference group = Male. ^d^ ADHD-RS-5 score.

**Table S4.** (continued).

| Transfer Range / Parameter | Immediately Post-intervention | | |  | 6 months Post-intervention | | |
| --- | --- | --- | --- | --- | --- | --- | --- |
|  | Est. [95% CI] | *p* | *f* ^2^ |  | Est. [95% CI] | *p* | *f* ^2^ |
| **Far transfer – hyperactivity^c^** |  |  |  |  |  |  |  |
| Intercept | 1.4[0.16,2.64] | .027 |  |  | 0.92[-0.74,2.58] | .273 |  |
| Baseline scores | 0.77[0.66,0.87] | <.001 | 1.96 |  | 0.76[0.64,0.89] | <.001 | 1.95 |
| Stratified age groups |  |  | 0.02 |  |  |  | 0.02 |
| 9-10 years vs. 7-8 years | -0.58[-1.46,0.3] | .194 |  |  | 0.4[-0.68,1.48] | .465 |  |
| 11-12 years vs. 7-8 years | -0.62[-2.23,0.99] | .447 |  |  | -0.52[-2.35,1.31] | .574 |  |
| Condition |  |  | 0.09 |  |  |  | 0.02 |
| Adaptive vs. active control | -2.25[-3.76,-0.75] | .004 |  |  | -0.93[-2.74,0.88] | .310 |  |
| Self-select vs. active control | -1.04[-2.79,0.71] | .240 |  |  | -1.33[-3.96,1.31] | .319 |  |
| Stepwise vs. active control | -0.89[-2.4,0.62] | .246 |  |  | -0.69[-2.57,1.2] | .470 |  |
| *Sex^a^* |  |  | *0.01* |  |  |  | *0.00* |
| *Female vs. male* | *-0.96[-2.56,0.64]* | *.236* |  |  | *-0.38[-2.5,1.74]* | *.721* |  |
| *Condition*Sex* |  |  | *0.07* |  |  |  | *0.00* |
| *Adaptive*Female* | *2.82[0.53,5.11]* | *.016* |  |  | *0.33[-2.44,3.11]* | *.812* |  |
| *Self-select*Female* | *2.33[-0.14,4.8]* | *.064* |  |  | *0.53[-2.85,3.9]* | *.757* |  |
| *Stepwise*Female* | *1.68[-0.56,3.91]* | *.140* |  |  | *0.41[-2.41,3.24]* | *.771* |  |
| Overall model | *F*(10, 100) = 24.07, *p* <.001, R^2^ = 0.71 | | | | *F*(10, 75) = 18.02, *p* < .001, R^2^ = 0.71 | | |

^a^ Reference group = Male

^d^ ADHD-RS-5 score.

**Table S5.** Exploratory ancillary analyses: comparisons from baseline to immediately post-intervention, and baseline to 6-months post-intervention while exploring differential effects of child age (relevant rows are in italics).

| Transfer Range / Parameter | Immediately Post-intervention | | |  | 6 months Post-intervention | | |
| --- | --- | --- | --- | --- | --- | --- | --- |
|  | Est. [95% CI] | *p* | *f* ^2^ |  | Est. [95% CI] | *p* | *f* ^2^ |
| **Near transfer - backward span digits** |  |  |  |  |  |  |  |
| Intercept | 2.11[0.51,3.71] | .010 |  |  | 4.62[2.83,6.41] | <.001 |  |
| Baseline scores | 0.58[0.42,0.74] | <.001 | 0.30 |  | 0.42[0.25,0.59] | <.001 | 0.13 |
| Condition |  |  | 0.01 |  |  |  | 0.00 |
| Adaptive vs. active control | -0.68[-2.53,1.17] | .468 |  |  | -0.56[-2.66,1.53] | .596 |  |
| Self-select vs. active control | 0.63[-1.22,2.48] | .503 |  |  | -0.3[-2.39,1.8] | .780 |  |
| Stepwise vs. active control | 0.13[-1.68,1.94] | .885 |  |  | -0.42[-2.44,1.6] | .684 |  |
| Stratified age groups |  |  | 0.02 |  |  |  | 0.01 |
| 9-10 years vs. 7-8 years | 1.35[-0.46,3.17] | .142 |  |  | 1.42[-0.6,3.45] | .167 |  |
| 11-12 years vs. 7-8 years | 3.73[-2.63,10.09] | .249 |  |  | 2.37[-2.74,7.48] | .361 |  |
| *Conditon*Stratified age groups* |  |  | *0.03* |  |  |  | *0.01* |
| *Adaptive*9-10 years* | *1.13[-1.41,3.67]* | *.382* |  |  | *0.4[-2.43,3.23]* | *.779* |  |
| *Self-select*9-10 years* | *-0.97[-3.74,1.8]* | *.490* |  |  | *-0.51[-3.61,2.59]* | *.745* |  |
| *Stepwise*9-10 years* | *-0.28[-2.83,2.28]* | *.832* |  |  | *0.11[-2.75,2.97]* | *.940* |  |
| *Adaptive*11-12 years* | *-1.5[-8.55,5.55]* | *.676* |  |  | *1.11[-4.88,7.09]* | *.716* |  |
| *Stepwise*11-12 years* | *1.11[-6.32,8.54]* | *.769* |  |  | *-2.31[-8.95,4.34]* | *.495* |  |
| Overall model | *F*(11, 179) = 8.45, *p* <.001, R^2^ = 0.34 | | |  | *F*(11, 183) = 4.58, *p* <.001, R^2^ = 0.22 | | |

**Table S5.** (continued).

| Transfer Range / Parameter | Immediately Post-intervention | | |  | 6 months Post-intervention | | |
| --- | --- | --- | --- | --- | --- | --- | --- |
|  | Est. [95% CI] | *p* | *f* ^2^ |  | Est. [95% CI] | *p* | *f* ^2^ |
| **Near transfer - following instructions objects** | |  |  |  |  |  |  |
| Intercept | 3.22[2.05,4.39] | <.001 |  |  | 5.01[3.64,6.39] | <.001 |  |
| Baseline scores | 0.32[0.17,0.46] | <.001 | 0.11 |  | 0.32[0.15,0.49] | <.001 | 0.08 |
| Condition |  |  | 0.03 |  |  |  | 0.01 |
| Adaptive vs. active control | 0.36[-0.84,1.57] | .556 |  |  | -0.74[-2.15,0.67] | .303 |  |
| Self-select vs. active control | 1.34[0.13,2.55] | .030 |  |  | -0.33[-1.75,1.08] | .645 |  |
| Stepwise vs. active control | 0.97[-0.21,2.16] | .107 |  |  | -0.45[-1.83,0.92] | .516 |  |
| Stratified age groups |  |  | 0.05 |  |  |  | 0.00 |
| 9-10 years vs. 7-8 years | 1.81[0.63,2.99] | .003 |  |  | 0.12[-1.25,1.49] | .864 |  |
| 11-12 years vs. 7-8 years | 2.25[-1.88,6.37] | .284 |  |  | 1.58[-1.88,5.04] | .369 |  |
| *Conditon*Stratified age groups* |  |  | *0.02* |  |  |  | *0.03* |
| *Adaptive*9-10 years* | *-0.14[-1.81,1.53]* | *.869* |  |  | *1.23[-0.69,3.15]* | *.206* |  |
| *Self-select*9-10 years* | *-1.39[-3.21,0.43]* | *.135* |  |  | *1.8[-0.32,3.92]* | *.095* |  |
| *Stepwise*9-10 years* | *-1.03[-2.7,0.64]* | *.227* |  |  | *1.09[-0.84,3.03]* | *.267* |  |
| *Adaptive*11-12 years* | *-0.34[-4.86,4.19]* | *.884* |  |  | *1.45[-2.57,5.47]* | *.477* |  |
| *Stepwise*11-12 years* | *-0.01[-4.81,4.8]* | *.998* |  |  | *-1.4[-5.88,3.08]* | *.539* |  |
| Overall model | *F*(11, 178) = 5.94, *p* <.001, R^2^ = 0.27 | | |  | *F*(11, 182) = 4.72, *p* <.001, R^2^ = 0.22 | | |

**Table S5.** (continued).

| Transfer Range / Parameter | Immediately Post-intervention | | |  | 6 months Post-intervention | | |
| --- | --- | --- | --- | --- | --- | --- | --- |
|  | Est. [95% CI] | *p* | *f* ^2^ |  | Est. [95% CI] | *p* | *f* ^2^ |
| **Near transfer - backward span letters** |  |  |  |  |  |  |  |
| Intercept | 2.92[1.77,4.08] | <.001 |  |  | 4.14[2.86,5.42] | <.001 |  |
| Baseline scores | 0.48[0.33,0.63] | <.001 | 0.23 |  | 0.32[0.16,0.48] | <.001 | 0.08 |
| Condition |  |  | 0.04 |  |  |  | 0.01 |
| Adaptive vs. active control | -1.36[-2.72,0] | .050 |  |  | 0.11[-1.38,1.61] | .881 |  |
| Self-select vs. active control | 0.47[-0.89,1.83] | .495 |  |  | 0.62[-0.87,2.12] | .412 |  |
| Stepwise vs. active control | 0.2[-1.13,1.53] | .766 |  |  | -0.29[-1.74,1.16] | .696 |  |
| Stratified age groups |  |  | 0.02 |  |  |  | 0.02 |
| 9-10 years vs. 7-8 years | 0.84[-0.5,2.18] | .217 |  |  | 0.33[-1.13,1.79] | .657 |  |
| 11-12 years vs. 7-8 years | 4.15[-0.5,8.8] | .080 |  |  | 3.25[-0.4,6.9] | .081 |  |
| *Conditon*Stratified age groups* |  |  | *0.10* |  |  |  | *0.01* |
| *Adaptive*9-10 years* | *2.2[0.34,4.06]* | *.021* |  |  | *0.06[-1.95,2.08]* | *.950* |  |
| *Self-select*9-10 years* | *-1.7[-3.73,0.34]* | *.101* |  |  | *0.39[-1.82,2.61]* | *.727* |  |
| *Stepwise*9-10 years* | *0[-1.88,1.89]* | *.997* |  |  | *0.61[-1.44,2.66]* | *.558* |  |
| *Adaptive*11-12 years* | *0.83[-4.29,5.96]* | *.749* |  |  | *-0.52[-4.78,3.75]* | *.811* |  |
| *Stepwise*11-12 years* | *-2.85[-8.28,2.57]* | *.301* |  |  | *-1.27[-6.02,3.48]* | *.598* |  |
| Overall model | *F*(11, 179) = 10.08, *p* <.001, R^2^ = 0.38 | | |  | *F*(11, 183) = 3.58, *p* <.001, R^2^ = 0.18 | | |

**Table S5.** (continued).

| Transfer Range / Parameter | Immediately Post-intervention | | |  | 6 months Post-intervention | | |
| --- | --- | --- | --- | --- | --- | --- | --- |
|  | Est. [95% CI] | *p* | *f* ^2^ |  | Est. [95% CI] | *p* | *f* ^2^ |
| **Near transfer - following instructions letters** | |  |  |  |  |  |  |
| Intercept | 3.42[2.22,4.62] | <.001 |  |  | 4.32[3.14,5.5] | <.001 |  |
| Baseline scores | 0.36[0.19,0.53] | <.001 | 0.10 |  | 0.28[0.11,0.44] | .001 | 0.06 |
| Condition |  |  | 0.00 |  |  |  | 0.01 |
| Adaptive vs. active control | -0.12[-1.32,1.08] | .845 |  |  | -0.26[-1.43,0.91] | .662 |  |
| Self-select vs. active control | -0.32[-1.51,0.87] | .598 |  |  | -0.55[-1.72,0.62] | .359 |  |
| Stepwise vs. active control | -0.36[-1.52,0.81] | .544 |  |  | -0.81[-1.95,0.32] | .159 |  |
| Stratified age groups |  |  | 0.01 |  |  |  | 0.00 |
| 9-10 years vs. 7-8 years | 0.78[-0.4,1.95] | .194 |  |  | 0[-1.15,1.14] | .998 |  |
| 11-12 years vs. 7-8 years | 0.07[-3.94,4.07] | .973 |  |  | 1.15[-1.67,3.97] | .421 |  |
| *Conditon*Stratified age groups* |  |  | *0.01* |  |  |  | *0.03* |
| *Adaptive*9-10 years* | *0.06[-1.56,1.67]* | *.946* |  |  | *0.93[-0.63,2.49]* | *.242* |  |
| *Self-select*9-10 years* | *0.11[-1.67,1.88]* | *.906* |  |  | *1.86[0.14,3.59]* | *.035^a^* |  |
| *Stepwise*9-10 years* | *-0.13[-1.77,1.52]* | *.878* |  |  | *0.57[-1.03,2.18]* | *.483* |  |
| *Adaptive*11-12 years* | *0.85[-3.54,5.24]* | *.704* |  |  | *-0.44[-3.72,2.83]* | *.790* |  |
| *Stepwise*11-12 years* | *2.36[-2.3,7.02]* | *.319* |  |  | *-0.94[-4.6,2.73]* | *.615* |  |
| Overall model | *F*(11, 176) = 3.28, *p* <.001, R^2^ = 0.17 | | |  | *F*(11, 181) = 3.00, *p* = .001, R^2^ = 0.15 | | |

^a^ Post-hoc analysis with Tukey’s HSD test to adjust for multiple comparisons not reveal any significant (p <.05) pairwise comparisons between conditions for the age groups.

**Table S5.** (continued).

| Transfer Range / Parameter | Immediately Post-intervention | | |  | 6 months Post-intervention | | |
| --- | --- | --- | --- | --- | --- | --- | --- |
|  | Est. [95% CI] | *p* | *f* ^2^ |  | Est. [95% CI] | *p* | *f* ^2^ |
| **Intermediate transfer - 2-back objects** |  |  |  |  |  |  |  |
| Intercept | 1.08[0.71,1.45] | <.001 |  |  | 1.65[1.31,1.98] | <.001 |  |
| Baseline scores | 0.18[0.04,0.32] | .012 | 0.04 |  | 0.21[0.08,0.33] | .001 | 0.06 |
| Condition | -0.08[-0.57,0.41] | .741 | 0.02 |  |  |  | 0.03 |
| Adaptive vs. active control |  |  |  |  | -0.13[-0.57,0.31] | .547 |  |
| Self-select vs. active control | -0.05[-0.54,0.44] | .835 |  |  | -0.37[-0.81,0.07] | .099 |  |
| Stepwise vs. active control | 0.28[-0.2,0.76] | .247 |  |  | -0.44[-0.86,-0.01] | .044 |  |
| Stratified age groups |  |  | 0.04 |  |  |  | 0.00 |
| 9-10 years vs. 7-8 years | 0.62[0.13,1.11] | .013 |  |  | -0.1[-0.53,0.33] | .653 |  |
| 11-12 years vs. 7-8 years | 1.37[-0.31,3.04] | .109 |  |  | 0.35[-0.74,1.43] | .529 |  |
| *Conditon*Stratified age groups* |  |  | *0.02* |  |  |  | *0.04* |
| *Adaptive*9-10 years* | *0.08[-0.59,0.76]* | *.807* |  |  | *0.21[-0.39,0.8]* | *.493* |  |
| *Self-select*9-10 years* | *-0.12[-0.86,0.61]* | *.742* |  |  | *0.69[0.04,1.34]* | *.038 ^a^* |  |
| *Stepwise*9-10 years* | *-0.41[-1.09,0.27]* | *.239* |  |  | *0.66[0.06,1.26]* | *.032 ^a^* |  |
| *Adaptive*11-12 years* | *-0.94[-2.78,0.89]* | *.312* |  |  | *-0.26[-1.51,0.99]* | *.684* |  |
| *Stepwise*11-12 years* | *-0.82[-2.77,1.14]* | *.410* |  |  | *-0.08[-1.48,1.32]* | *.908* |  |
| Overall model | *F*(11, 178) = 3.406, *p* <.001, R^2^ = 0.17 | | |  | *F*(11, 183) = 2.62, *p* = .004, R^2^ = 0.14 | | |

^a^ Post-hoc analysis with Tukey’s HSD test to adjust for multiple comparisons not reveal any significant (p <.05) pairwise comparisons between conditions for the age groups.

**Table S5.** (continued).

| Transfer Range / Parameter | Immediately Post-intervention | | |  | 6 months Post-intervention | | |
| --- | --- | --- | --- | --- | --- | --- | --- |
|  | Est. [95% CI] | *p* | *f* ^2^ |  | Est. [95% CI] | *p* | *f* ^2^ |
| **Far transfer – reasoning^b^** |  |  |  |  |  |  |  |
| Intercept | 4.95[3.04,6.86] | <.001 |  |  | 9.37[7.3,11.43] | <.001 |  |
| Baseline scores | 0.74[0.64,0.84] | <.001 | 1.17 |  | 0.55[0.45,0.66] | <.001 | 0.56 |
| Condition |  |  | 0.03 |  |  |  | 0.02 |
| Adaptive vs. active control | 1.33[0.09,2.57] | .036 |  |  | 0.11[-1.2,1.42] | .868 |  |
| Self-select vs. active control | 0.77[-0.47,2.01] | .220 |  |  | 0.93[-0.39,2.24] | .166 |  |
| Stepwise vs. active control | 0.45[-0.76,1.66] | .466 |  |  | -0.21[-1.48,1.06] | .746 |  |
| Stratified age groups |  |  | 0.01 |  |  |  | 0.00 |
| 9-10 years vs. 7-8 years | 0.46[-0.77,1.69] | .459 |  |  | -0.53[-1.82,0.75] | .416 |  |
| 11-12 years vs. 7-8 years | 2.32[-1.92,6.56] | .282 |  |  | -0.2[-3.43,3.02] | .901 |  |
| *Conditon*Stratified age groups* |  |  | *0.02* |  |  |  | *0.02* |
| *Adaptive*9-10 years* | *-1.15[-2.84,0.55]* | *.183* |  |  | *0.51[-1.25,2.28]* | *.566* |  |
| *Self-select*9-10 years* | *-0.52[-2.37,1.33]* | *.581* |  |  | *-0.3[-2.25,1.64]* | *.759* |  |
| *Stepwise*9-10 years* | *-0.15[-1.86,1.57]* | *.866* |  |  | *-0.27[-2.07,1.53]* | *.766* |  |
| *Adaptive*11-12 years* | *-2.93[-7.58,1.72]* | *.215* |  |  | *-0.06[-3.79,3.67]* | *.975* |  |
| *Stepwise*11-12 years* | *-1.54[-6.48,3.4]* | *.540* |  |  | *2.17[-2,6.34]* | *.306* |  |
| Overall model | *F*(11, 179) = 24.76, *p* <.001, R^2^ = 0.60 | | |  | *F*(11, 183) = 11.74, *p* < .001, R^2^ = 0.41 | | |

^b^ Raven’s SPM sets A and B score.

**Table S5.** (continued).

| Transfer Range / Parameter | Immediately Post-intervention | | |  | 6 months Post-intervention | | |
| --- | --- | --- | --- | --- | --- | --- | --- |
|  | Est. [95% CI] | *p* | *f* ^2^ |  | Est. [95% CI] | *p* | *f* ^2^ |
| **Far transfer – inattention^c^** |  |  |  |  |  |  |  |
| Intercept | 1.89[0.51,3.27] | .008 |  |  | 0.63[-1.13,2.39] | .478 |  |
| Baseline scores | 0.77[0.68,0.85] | <.001 | 3.32 |  | 0.73[0.63,0.83] | <.001 | 3.04 |
| Condition |  |  | 0.01 |  |  |  | 0.03 |
| Adaptive vs. active control | 0.24[-1.55,2.03] | .792 |  |  | 1.43[-0.71,3.57] | .187 |  |
| Self-select vs. active control | 0.27[-1.59,2.13] | .774 |  |  | 0.03[-2.16,2.22] | .978 |  |
| Stepwise vs. active control | -0.61[-2.41,1.18] | .498 |  |  | 0.92[-1.28,3.12] | .406 |  |
| Stratified age groups |  |  | 0.01 |  |  |  | 0.04 |
| 9-10 years vs. 7-8 years | -0.19[-2.02,1.63] | .834 |  |  | 1.51[-0.78,3.81] | .193 |  |
| 11-12 years vs. 7-8 years | 1.69[-1.86,5.23] | .347 |  |  | -2.09[-7.21,3.03] | .419 |  |
| *Conditon*Stratified age groups* |  |  | *0.07* |  |  |  | *0.08* |
| *Adaptive*9-10 years* | *-0.88[-3.48,1.71]* | *.499* |  |  | *-3.55[-6.62,-0.49]* | *.024^a^* |  |
| *Self-select*9-10 years* | *-2.63[-5.4,0.13]* | *.061* |  |  | *-1.64[-5.24,1.96]* | *.368* |  |
| *Stepwise*9-10 years* | *-0.3[-2.88,2.27]* | *.817* |  |  | *-2.37[-5.47,0.74]* | *.133* |  |
| *Adaptive*11-12 years* | *-3.99[-8.42,0.44]* | *.077* |  |  | *-0.05[-5.87,5.78]* | *.987* |  |
| *Stepwise*11-12 years* | *-3.01[-7.65,1.62]* | *.200* |  |  | *0.92[-5.1,6.94]* | *.761* |  |
| Overall model | *F*(11, 99) = 35.80, *p* <.001, R^2^ = 0.80 | | |  | *F*(11, 74) = 25.76, *p* < .001, R^2^ = 0.80 | | |

^a^ Post-hoc analysis with Tukey’s HSD test to adjust for multiple comparisons not reveal any significant (p <.05) pairwise comparisons between conditions for the age groups.

^c^ ADHD-RS-5 score.

**Table S5.** (continued).

| Transfer Range / Parameter | Immediately Post-intervention | | |  | 6 months Post-intervention | | |
| --- | --- | --- | --- | --- | --- | --- | --- |
|  | Est. [95% CI] | *p* | *f* ^2^ |  | Est. [95% CI] | *p* | *f* ^2^ |
| **Far transfer – hyperactivity^d^** |  |  |  |  |  |  |  |
| Intercept | 0.36[-0.89,1.61] | .567 |  |  | 0.98[-0.61,2.56] | .225 |  |
| Baseline scores | 0.77[0.66,0.88] | <.001 | 1.89 |  | 0.77[0.64,0.89] | <.001 | 2.02 |
| Condition | -0.63[-2.3,1.05] | .461 | 0.05 |  |  |  | 0.03 |
| Adaptive vs. active control |  |  |  |  | -1.1[-3.07,0.88] | .272 |  |
| Self-select vs. active control | 1.19[-0.53,2.91] | .172 |  |  | -1.34[-3.32,0.63] | .180 |  |
| Stepwise vs. active control | 0.71[-0.94,2.36] | .395 |  |  | -0.89[-2.9,1.13] | .383 |  |
| Stratified age groups |  |  | 0.01 |  |  |  | 0.00 |
| 9-10 years vs. 7-8 years | 0.72[-0.97,2.41] | .398 |  |  | -0.1[-2.23,2.02] | .922 |  |
| 11-12 years vs. 7-8 years | 1.26[-2.08,4.59] | .457 |  |  | -0.98[-5.76,3.81] | .686 |  |
| *Conditon*Stratified age groups* |  |  | *0.04* |  |  |  | *0.02* |
| *Adaptive*9-10 years* | *-0.7[-3.13,1.73]* | *.569* |  |  | *0.9[-1.96,3.75]* | *.533* |  |
| *Self-select*9-10 years* | *-2.19[-4.75,0.37]* | *.093* |  |  | *0.65[-2.63,3.94]* | *.693* |  |
| *Stepwise*9-10 years* | *-1.5[-3.87,0.86]* | *.210* |  |  | *0.53[-2.33,3.39]* | *.713* |  |
| *Adaptive*11-12 years* | *-1.64[-5.8,2.52]* | *.435* |  |  | *-0.21[-5.65,5.24]* | *.940* |  |
| *Stepwise*11-12 years* | *-2.94[-7.28,1.4]* | *.182* |  |  | *1.61[-4,7.21]* | *.570* |  |
| Overall model | *F*(11, 99) = 20.31, *p* <.001, R^2^ = 0.69 | | |  | *F*(11, 74) = 16.61, *p* < .001, R^2^ = 0.71 | | |

^c^ ADHD-RS-5 score.
